# SIRT2 is associated with prognosis, ferroptosis susceptibility, and immune infiltration in lung adenocarcinoma

**DOI:** 10.3389/fonc.2026.1900448

**Published:** 2026-09-11

**Authors:** Wei Zhang, Li-sha Ren, Huan Bai, Ying-ying Mu, Yue Qian, Hongyu Chen

**Affiliations:** 1 Medical Affairs Department, The Second Affiliated Hospital of Zunyi Medical University, Zunyi, Guizhou, China; 2 Department of Nephrology, The Second Medical Center of PLA General Hospital, National Clinical Research Center for Geriatric Diseases, Beijing, China; 3 Department of Pathology, Zunyi Hospital of Traditional Chinese Medicine, Zunyi, Guizhou, China; 4 Department of Pathogen Biology, Guizhou Nursing Vocational College, Guiyang, Guizhou, China

**Keywords:** biomarkers, ferroptosis, lung adenocarcinoma, prognosis, SIRT2, tumor-immune infiltration

## Abstract

**Objective:**

Lung adenocarcinoma (LUAD) is a highly aggressive malignancy originating from the bronchial epithelium or glandular tissues and represents the most rapidly increasing subtype of lung cancer worldwide. Its high incidence and mortality rates contribute to an overall unfavorable prognosis. Therapeutic options for patients with advanced-stage LUAD remain limited, emphasizing the urgent importance of identifying reliable prognostic biomarkers and novel therapeutic targets. Silent information regulator 2 (SIRT2), a member of the mammalian sirtuin family, has been implicated in multiple cancer types; however, its functional relevance in LUAD has not yet been comprehensively defined.

**Methods:**

Data from TCGA and GEO were employed to evaluate SIRT2 expression patterns in LUAD. Functional enrichment approaches were applied to infer the biological pathways potentially regulated by SIRT2. Molecular docking and Gene Set Cancer Analysis (GSCA) were used to examine how SIRT2 expression and drug sensitivity are related, and to identify potential small-molecule interactors. Key candidate targets and compounds were subsequently subjected to in silico docking validation. The bioinformatic predictions were further verified through in vitro functional assays and Western blotting using A549 LUAD cells.

**Results:**

SIRT2 expression was markedly downregulated in LUAD tissues, and low SIRT2 levels were closely associated with adverse clinical prognostic features, including pathological stage, gender and primary treatment response. Reduced SIRT2 expression correlated with poorer overall, progression-free, and disease-specific survival. Functional enrichment indicated that SIRT2 is involved in multiple core signaling pathways implicated in LUAD progression. Furthermore, SIRT2 expression was strongly associated with ferroptosis-related pathways and estimated immune cell infiltration. Experimental validation in A549 LUAD cells revealed that SIRT2 is linked to suppressed malignant cellular phenotypes and reduced expression of the ferroptosis suppressor GPX4.

**Conclusions:**

Collectively, these findings support SIRT2 as a potential prognostic biomarker for LUAD. The molecular networks and pathways uncovered here advance our understanding of SIRT2 biology and lay a foundation for further research. Additional studies focusing on immune-related signatures correlated with SIRT2 may facilitate the development of novel immunotherapeutic approaches for LUAD.

## 1 Introduction

Lung adenocarcinoma is the most common histological form of primary lung cancer, accounting for more than 40% of all cases worldwide, and its incidence continues to increase each year (1, 2). Lung cancer remains the leading cause of cancer-related death globally, with an estimated 2.2 million new cases and 1.8 million deaths annually. Epidemiological investigations have identified smoking, air pollution, and genetic susceptibility as major risk factors (3). Current treatment modalities for cancer include radiotherapy, chemotherapy, traditional Chinese medicine, surgical resection, and immunotherapy (4–7). Patients frequently present with clinical symptoms such as chronic cough and shortness of breath. From a pathological perspective, LUAD comprises a continuum of disease stages, ranging from preinvasive lesions, including adenocarcinoma *in situ* (AIS) (8) and minimally invasive adenocarcinoma (MIA) (9), to fully invasive adenocarcinoma (10) and its various histological variants. Current treatment strategies rely on a combination of surgical resection, chemotherapy, immunotherapy, and radiotherapy (11–13). Despite advances in clinical management, a major obstacle remains the high proportion of patients diagnosed at advanced stages, often accompanied by distant metastasis. Early detection is challenging, and many patients rapidly develop resistance to systemic therapies, which severely restricts long-term treatment efficacy and accelerates disease progression. As a result, the overall prognosis for LUAD is still dismal, with five-year survival rates remaining below 20% (14, 15). This underscores the urgent need to identify robust molecular biomarkers that can improve prognostic evaluation and support personalized therapeutic strategies for patients with LUAD.

Sirtuin 2 (SIRT2) is a member of the sirtuin family, which consists of seven NAD^+^-dependent lysine deacetylases (SIRT1–SIRT7) that regulate protein function by removing acetyl groups from histone and non-histone substrates (16). SIRT2 is localized in both the cytoplasm and nucleus, where it deacetylates α-tubulin and histone residues such as H4K16 and H4K20, thereby contributing to chromosome stability and proper mitotic progression (17). In lung adenocarcinoma, SIRT2 has been suggested to modulate metabolic reprogramming and epithelial-mesenchymal transition, but its precise molecular mechanisms remain poorly defined. Specifically, the link between SIRT2 and key LUAD molecular features (e.g., KRAS, TP53, and EGFR mutations) as well as clinical progression from preinvasive to invasive stages has not been fully explored. Increasing evidence suggests that SIRT2 plays important roles in tumor biology, including in breast cancer (18), colorectal cancer (19), and epithelial ovarian cancer (20). However, its expression pattern in LUAD and the mechanisms by which it may influence tumor invasion and metastasis remain poorly characterized.

To address these gaps, the present study systematically evaluated SIRT2 expression in LUAD and examined its associations with key clinicopathological features. How SIRT2 and immune cell infiltration, immune-related gene signatures, and ferroptosis are linked were also explored using several public bioinformatics platforms. By integrating pathway enrichment analyses with immune profiling, we sought to predict the functional involvement of SIRT2 in LUAD progression. Together, the objectives were clarification of the functions of SIRT2 in LUAD and its potential in treating and managing the disease.

## 2 Methods

### 2.1 Data

Pan-cancer information on survival, gene expression, and immune features was retrieved from the UCSC Xena TCGA Pan-Cancer database (21). The TCGA-LUAD cohort was used to analyze SIRT2 expression and its association with clinicopathological parameters. Two independent GEO datasets, GSE43458 and GSE32863, were also included for validation of SIRT2 levels in LUAD. Specifically, GSE43458 contains 80 LUAD and 30 normal lung tissue samples, while GSE32863 comprises 58 LUAD and 58 matched normal adjacent tissues. In addition, immunohistochemical (IHC) staining data for SIRT2 across 17 cancer types and 44 normal samples from the Human Protein Atlas (HPA) database were retrieved for analysis (22).

### 2.2 Prognostic analysis

Univariate Cox proportional hazards regression was undertaken to assess the link between SIRT2 levels and overall survival (OS), disease-specific survival (DSS), and the progression-free interval (PFI) across multiple cancer types, including STAD, COAD, HNSC, LUSC, LIHC, KIRC, KICH, CHOL, and THCA. The results were visualized using forest plots displaying hazard ratios (HRs), 95% confidence intervals (CIs), and corresponding P-values using the R “forestplot” package (23). Kaplan-Meier survival curves based on SIRT2 expression levels in LUAD samples were constructed using the “survival” and “survminer” packages in R.

### 2.3 Genetic alteration analysis

The frequency and types of SIRT2 mutations, copy number variations, and genomic alterations across LUAD cohorts were analyzed using cBioPortal (https://www.cbioportal.org/) (24). Mutation categories and distributions were further examined through the COSMIC database.

### 2.4 Functional enrichment analysis

Differential expression analysis of SIRT2 in the TCGA-LUAD dataset was performed using the limma package in R. Genes with |log_2_FC| > 1 and FDR-adjusted p < 0.05 (Benjamini-Hochberg method) were considered differentially expressed. Pearson correlation analysis was used to identify genes significantly co-expressed with SIRT2 (|r| > 0.3, p < 0.05). To explore the biological processes associated with SIRT2, TCGA-LUAD expression data were used to identify genes significantly co-expressed with SIRT2. Gene functions were assessed using GO (December 2023) and KEGG (25) (Release 104.0, December 2023) analyses via GEPIA2.0, which integrates the clusterProfiler package for enrichment computations (26). KEGG database was used under permission from Kanehisa Laboratories (https://www.kegg.jp/kegg/kegg1.html).

### 2.5 Immune cell infiltration analyses

A deconvolution-based strategy was employed to assess the association between SIRT2 levels and the proportions of immune cell types in LUAD. Immune cells were examined using the TIMER platform and TCGA transcriptomic data, enabling quantification of major immune cell populations, such as macrophages, neutrophils, and B, CD8^+^ T, CD4^+^ T, and dendritic cells (27). Least squares regression was applied to compare immune cell-specific gene signatures with predefined reference matrices. Standardized TIMER parameters ensured analytical consistency. Correlations between SIRT2 expression and immune-related signatures were further analyzed via ssGSEA. Data visualization was performed with ggplot2 (v3.4.4).

### 2.6 Drug sensitivity analysis

Associations between SIRT2 expression and drug response were evaluated using the GSCA platform (28) and the Genomics of Drug Sensitivity in Cancer (GDSC) database. Pearson correlations were utilized to determine relationships between SIRT2 mRNA levels and drug IC50 values, with Benjamini-Hochberg false discovery rate (FDR) adjustments applied to p-values.

### 2.7 Immunohistochemistry

IHC staining was performed on LUAD tissue microarray sections (5 μm thickness; cat. no. SP103, Expectlab, Qingdao, Shandong, China) following standard protocols (29). After deparaffinization, rehydration, antigen retrieval, and blocking (5% goat serum and 3% H_2_O_2_), sections were treated overnight with mouse anti-human SIRT2 antibody (1:200; cat. no. K001382M, Beijing Solarbio Science & Technology™, China) at 4 °C. Signal detection was carried out using AEC^®^ substrate (cat. no. ZLI-9036, Beijing Zhongshan Jinqiao Biotechnology™). Staining intensity was quantified using Image-Tool II^®^ (University of Texas, USA) with grayscale values ranging from 0 to 255.

### 2.8 Molecular docking of Epothilone B with SIRT2

Molecular docking simulations were performed using Schrödinger Suite Maestro (v11.5) to predict binding interactions between SIRT2 and candidate ligands. Structures were obtained from the Protein Data Bank, SWISS-MODEL, and PubChem. Docking results were visualized and processed following previously established protocols (30). PyMOL (v2.3) was used to analyze three-dimensional binding conformations and calculate affinity scores.

### 2.9 Cell culture

A549 cells (CL-0016; Wuhan, China) were grown in DMEM with 10% FBS at 37 °C and 5% CO_2_.

### 2.10 Transfection

An overexpression plasmid for SIRT2 (OE-SIRT2) and a negative control vector (NC-SIRT2) (31) were procured from Gima Gene (Shanghai, China). The vector is driven by a CMV promoter and contains the full-length human SIRT2 coding sequence (NM_012237.4). Transfection was performed using Lipofectamine 6000 (Beyotime Biotechnology) for 48 h. All experiments were performed in quadruplicate.

### 2.11 Western immunoblotting

Total protein was extracted from LUAD cells using RIPA buffer (P0013B, Beyotime Biotechnology) with PMSF (ST506) and phosphatase inhibitors (P1081), followed by SDS-PAGE separation and transfer to PVDF membranes (0.45 μm; Merck Millipore, USA). Equal total protein (20 μg) was loaded into each well, and GAPDH was used as the internal reference to verify consistent sample loading across all groups. After blocking (5% BSA, 1 h), blots were treated with mouse anti-SIRT2 (1:1000; K001382M, Solarbio), Rabbit anti-NRF2 (1:1000; AF7623, beyotime), mouse anti-GPX4 (1:1000; K010078M, Solarbio), and rabbit anti-GAPDH (1:2000; K110496P, Solarbio) antibodies, followed by appropriate secondary antibodies and visualization with a 2500B imaging system (Tanon, Shanghai, China) and quantification with ImageJ (v1.6).

### 2.12 CCK-8 assays

CCK-8 assays (cat. no. CA1210, Solarbio) were used when assessing proliferation. Transfected cells (5 × 10^3^/well) were inoculated into 96-well plates, with six technical replicates for each treatment. After 48 h of incubation, 10 μL of CCK-8 solution was introduced to each well for 1 h at 37 °C, after which absorbances at 450 nm were read in a microplate reader to quantify cell viability and growth inhibition.

### 2.13 Wound healing assay

At 48 h post-transfection, A549 cells were grown in 6-well culture dishes until 90% confluent, after which a linear scratch was made in the monolayer using a sterile 10 μL pipette tip. After washing away floating cells, the remainder were grown in serum-free DMEM medium to block cell proliferation and exclude proliferative interference for migration statistics. Wounds were imaged at 0, 24, and 48 h to assess cell migratory capacity. No mitosis inhibitor was applied, as serum starvation sufficiently suppressed cell division.

### 2.14 Transwell assays

Cells (3 × 10^4^ in 100 μL of serum-free DMEM) were placed in the top compartment of a Transwell insert. The bottom compartment contained 600 μL of DMEM with 10% FBS. Following 48 h at 37 °C, non-migrated cells were removed, and the migrated cells were fixed and stained with crystal violet solution (C0121, Beyotime Biotechnology). For quantification, five non-overlapping random visual fields under 200× light microscope were captured and counted for each biological replicate; the counted cells were defined as invasive metastatic cells per field. Stained cells were visualized and counted under a light microscope.

### 2.15 Statistical analyses

Bioinformatic data analyses were performed using R (v4.0.3). Quantitative data are expressed as mean ± SEM. Prior to parametric tests, normality was assessed using the Shapiro–Wilk test and homogeneity of variances using Levene’s test. For normally distributed data with equal variances, two-group comparisons were performed using Student’s t-test, and multi-group comparisons using one-way ANOVA followed by Tukey’s *post hoc* test. For non-normally distributed data, the Mann-Whitney U test or Kruskal-Wallis test was applied. Associations between SIRT2 levels and clinicopathological parameters were evaluated using Wilcoxon rank-sum tests, Fisher’s exact tests, chi-square tests, and logistic regression models. All p-values were two-sided, and p < 0.05 was considered statistically significant. For multiple comparisons, the Benjamini-Hochberg FDR method was applied, and adjusted q-values < 0.05 were considered significant.

## 3 Results

### 3.1 SIRT2 downregulation is evident across multiple cancers

**Figure 1:**
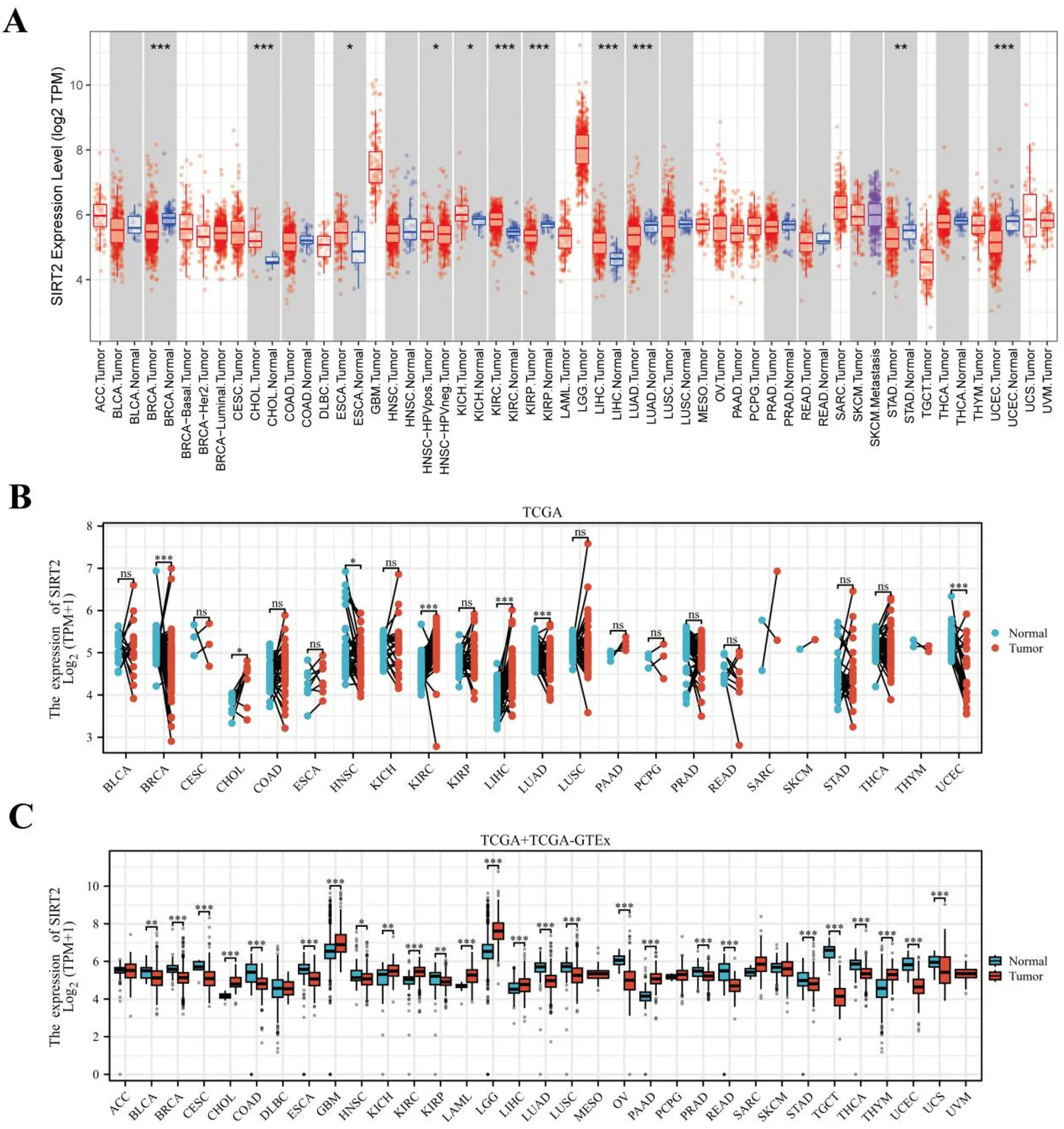
Pan-cancer profiling of SIRT2 levels. **(A)** SIRT2 levels in tumor and corresponding normal tissue samples from TCGA, based on TIMER 2.0. **(B)** SIRT2 expression in matched TCGA tumor and normal samples. **(C)** Comparison of SIRT2 expression between GTEx and TCGA datasets using Wilcoxon rank-sum tests. *p < 0.05; **p < 0.01; ***p < 0.001; ns, not significant. TPM, transcripts per million.

SIRT2 expression patterns were first examined across diverse malignancies using the TIMER 2.0 platform. Relative to normal tissues, SIRT2 was markedly downregulated in BRCA, KIRP, LUAD, STAD, and UCEC, with upregulation seen in CHOL, ESCA, KICH, KIRC, and LIHC (**Figure 1A**).

Analyses of matched tumor-normal pairs yielded comparable trends. Specifically, SIRT2 expression was reduced in BRCA, HNSC, LUAD, and UCEC, while elevated levels were found in CHOL, KIRC, and LIHC relative to adjacent normal tissues (**Figure 1B**). To further validate these findings, combined data from the GTEx and TCGA repositories were analyzed. This integrated approach demonstrated significantly lower SIRT2 expression in a wide range of tumor types, including LUAD, BLCA, TGCT, THCA, LUSC, OV, PRAD, READ, HNSC, KIRP, UCEC, CESC, COAD, ESCA, BRCA, STAD, and UCS, compared with normal tissues (**Figure 1C**).

### 3.2 SIRT2 levels are associated with prognostic outcomes

**Figure 2:**
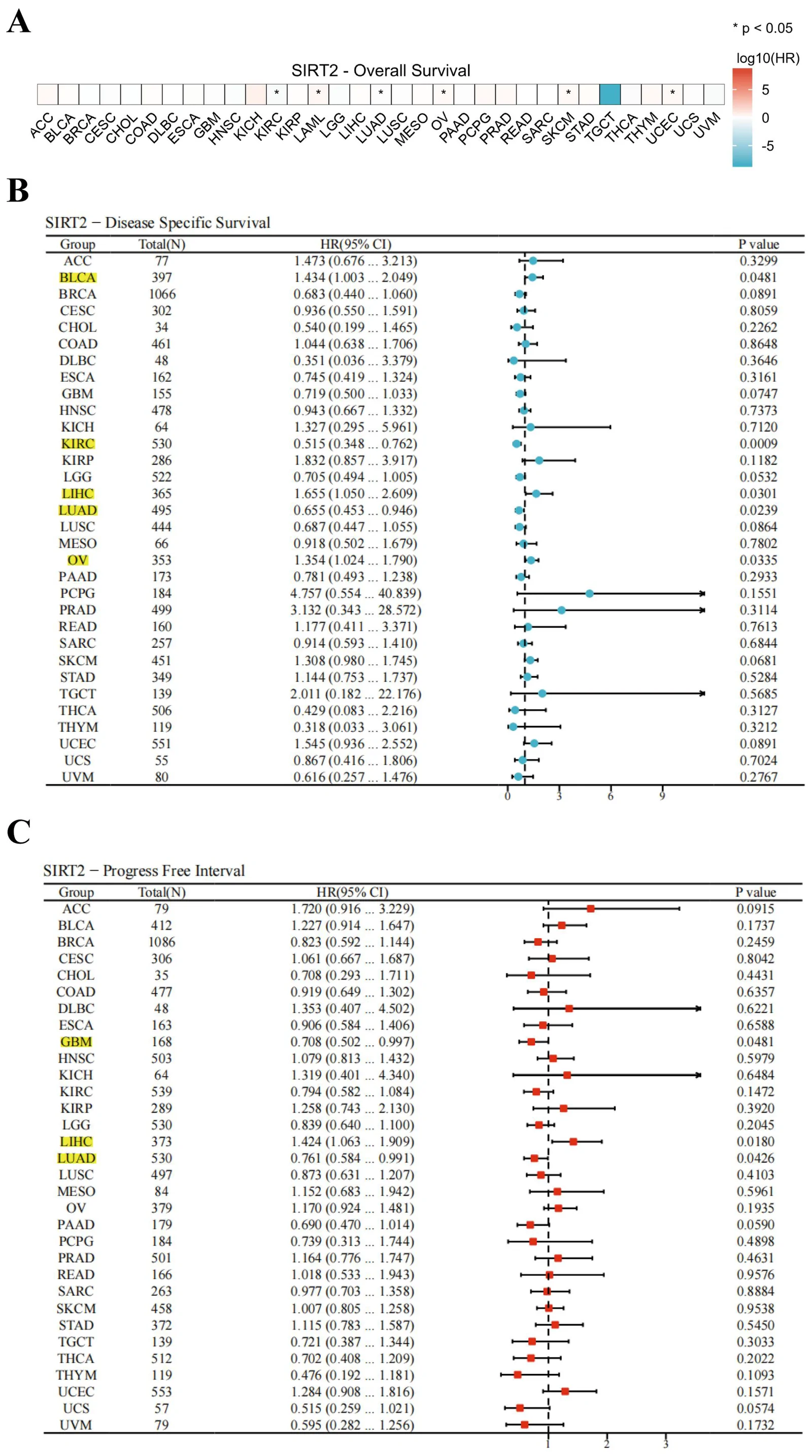
Analysis of survival in pan-cancer based on SIRT2 levels. **(A)** OS. **(B)** DSS. **(C)** PFI. *p < 0.05. All p-values were corrected using the Benjamini–Hochberg FDR method.

Differential expression analyses revealed significant associations between SIRT2 levels and prognostic outcomes across multiple cancer types, including BLCA, HNSC, LUAD, and UCEC. Based on these results, the prognostic relevance of SIRT2 was further examined through survival analyses (**Figure 2**). OS differed significantly according to SIRT2 expression in patients with KIRC, LAML, LUAD, OV, SKCM, and UCEC (**Figure 2A**), suggesting that SIRT2 levels influence patient outcomes in these cancers. Consistent trends were observed for disease-specific survival (DSS), in which SIRT2 expression was significantly associated with outcomes in BLCA, KIRC, LIHC, LUAD, and OV (**Figure 2B**). Lower SIRT2 levels were correlated with poorer DSS in LUAD (p = 0.0239; HR = 0.655), BLCA (p = 0.0481; HR = 1.434), and OV (p = 0.0335; HR = 1.354), whereas the opposite pattern was observed in KIRC (p = 0.0009; HR = 0.515) and LIHC (p = 0.0301; HR = 1.655). PFI analyses further demonstrated significant associations between SIRT2 expression and patient outcomes in glioblastoma multiforme (GBM; p = 0.0481; HR = 0.708), LIHC (p = 0.0180; HR = 1.424), and LUAD (p = 0.0426; HR = 0.761) (**Figure 2C**). Collectively, these findings indicate that SIRT2 expression is a robust predictor of clinical prognosis, particularly in LUAD, across multiple survival endpoints, underscoring its potential clinical relevance.

### 3.3 LUAD is characterized by SIRT2 downregulation

**Figure 3:**
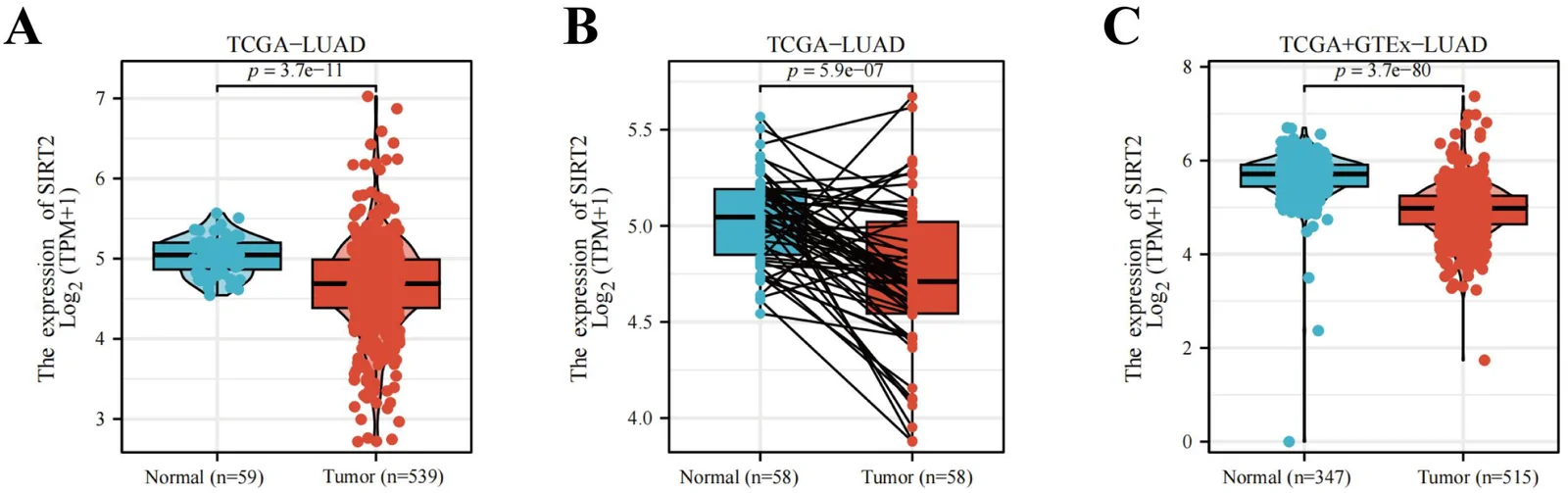
SIRT2 expression in the TCGA-LUAD cohort. **(A)** SIRT2 levels in TCGA-LUAD tumors versus normal tissue samples (p = 3.7 × 10^-^¹¹). **(B)** Analysis of SIRT2 levels in LUAD and adjacent tissues (p = 5.9 × 10^-7^). **(C)** SIRT2 levels in combined TCGA and GTEx datasets (p = 3.7 × 10^--0^).

To further clarify the role of SIRT2 in LUAD, expression data from the TCGA-LUAD dataset and GTEx were analyzed. SIRT2 levels were markedly lower in 539 LUAD tumor samples relative to 59 normal lung samples (p = 3.7 × 10^-11^; **Figure 3A**). Analysis of 58 paired tumor and adjacent non-tumor tissues confirmed this reduction (p = 5.9 × 10^-7^; **Figure 3B**). When TCGA and GTEx datasets were combined, SIRT2 mRNA levels were also markedly decreased in 515 tumor samples relative to 347 normal controls (p = 3.7 × 10^-80^; **Figure 3C**).

**Figure 4:**
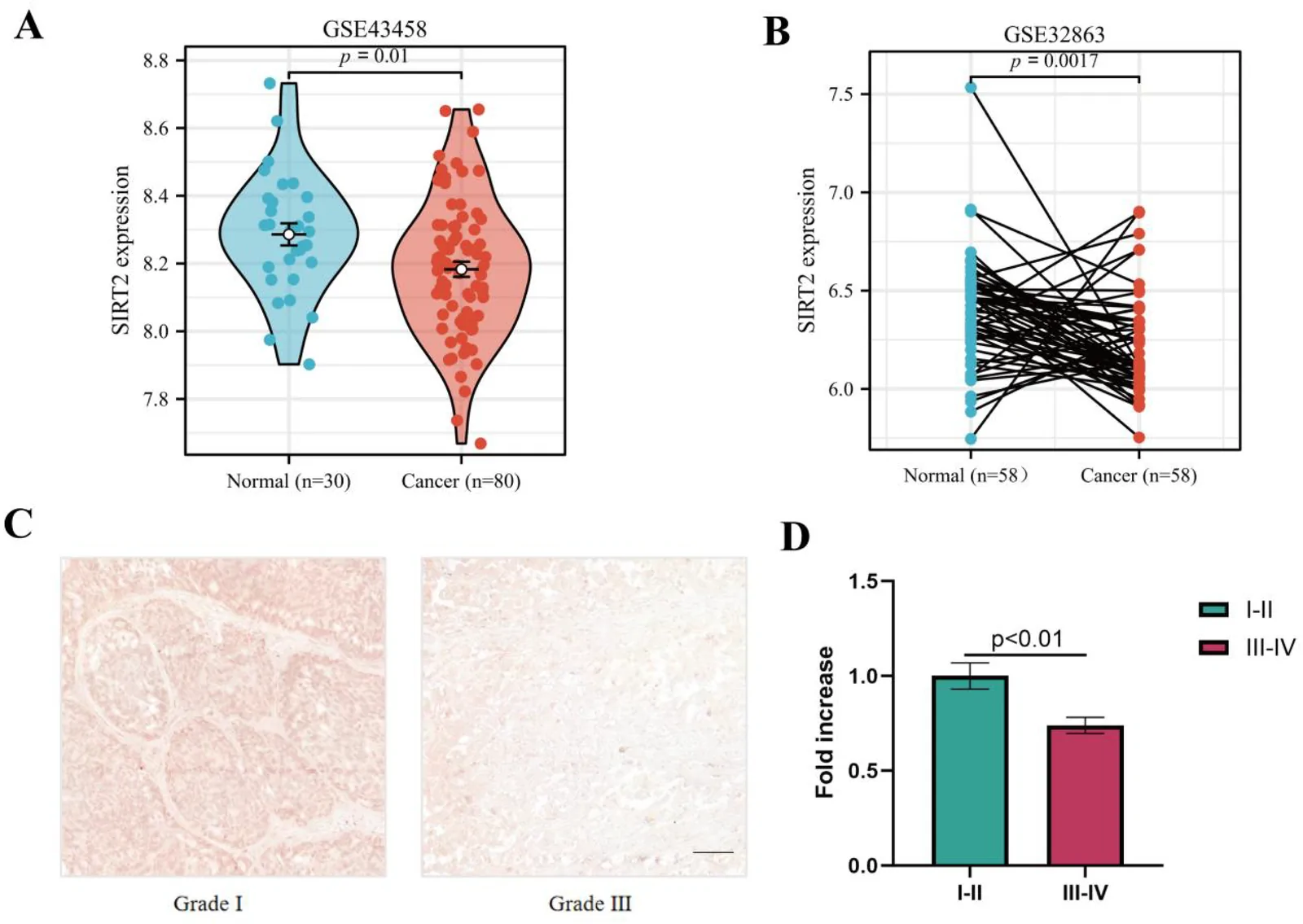
Validation of SIRT2 downregulation in LUAD. **(A)** SIRT2 expression in GSE43458. **(B)** SIRT2 expression in GSE32863. **(C)** Representative IHC images of SIRT2 in LUAD tissues (scale bar, 50 μm). **(D)** Quantitative comparison of SIRT2 staining intensity across tumor grades.

These observations were further validated using two independent GEO datasets. In GSE43458 (80 LUAD vs. 30 normal), SIRT2 expression was significantly lower in LUAD tissues than in controls (p = 0.01); similarly, in GSE32863 (58 LUAD vs. 58 normal) SIRT2 expression was reduced (p = 0.0017) (**Figures 4A, B**). To corroborate these transcriptomic findings at the protein level, immunohistochemical staining was performed on LUAD tissue samples. Analysis of 25 grade I-II and 22 grade III-IV tumors revealed significantly reduced SIRT2 expression in higher-grade specimens (p < 0.01) (**Figures 4C, D**). Together, these data provide compelling evidence that SIRT2 is consistently downregulated in LUAD and is associated with tumor progression.

### 3.4 Prognostic significance of SIRT2 in LUAD

**Figure 5:**
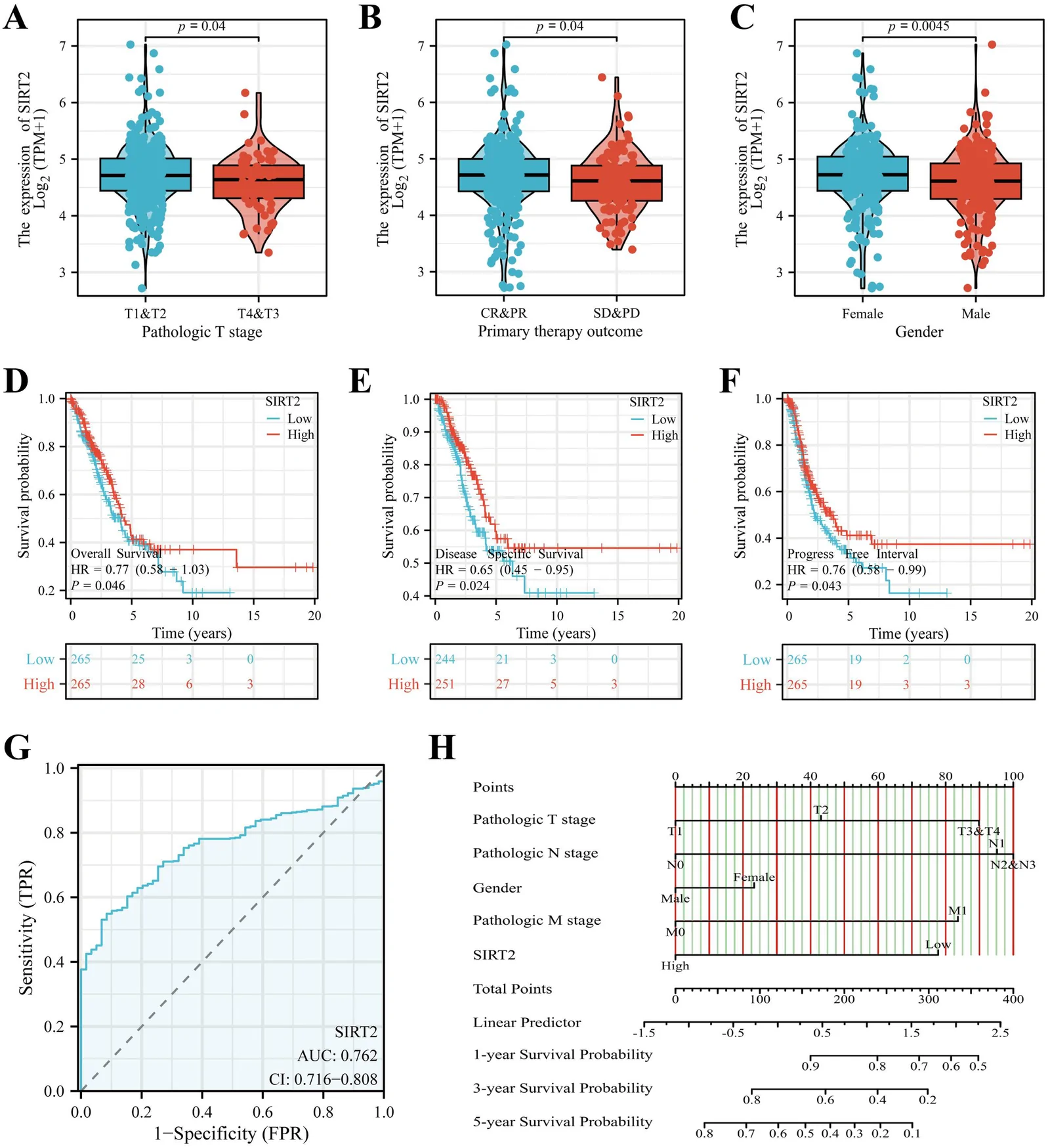
Association between SIRT2 expression, clinicopathological features, and survival in LUAD. **(A)** Correlation between SIRT2 expression and T stage. T1&T2: pooled T1/T2 early stages; T3&T4: pooled T3/T4 advanced stages. **(B)** Relationship between SIRT2 levels and primary therapy outcome. **(C)** Association between SIRT2 expression and sex. **(D–F)** Kaplan-Meier curves comparing OS **(D)**, DSS **(E)**, and PFI **(F)** between high- and low-SIRT2 cohorts (p < 0.05; red, high; blue, low). **(G)** ROC curve showing the diagnostic ability of SIRT2. **(H)** Nomogram predicting one-, three-, and five-year OS in LUAD cases.

Clinical data and SIRT2 levels from the TCGA LUAD cohort were jointly analyzed to explore potential prognostic relationships. Univariate analyses revealed that reduced SIRT2 levels showed marked linkage with tumor T stage, primary therapy outcome, and patient sex (all p < 0.05; **Figures 5A–C**). These findings show that LUAD patients with lower SIRT2 levels are more likely to present with more advanced disease compared with those exhibiting higher expression.

Kaplan-Meier curves further demonstrated that patients with low SIRT2 expression exhibited significantly poorer OS (HR = 0.77, p = 0.046; **Figure 5D**), DSS (HR = 0.65, p = 0.024; **Figure 5E**), and PFI (HR = 0.76, p = 0.043; **Figure 5F**) compared to those with high SIRT2 expression. The static ROC curve in **Figure 5G** revealed that SIRT2 possessed good diagnostic discriminatory power for LUAD, with an AUC of 0.762. To determine whether SIRT2 could independently predict survival after adjusting for clinical confounders, we performed multivariable Cox proportional hazards regression analysis incorporating T stage, N stage, gender, and SIRT2 expression (**Supplementary Figure 1A**). After adjustment, low SIRT2 expression remained an independent adverse prognostic factor for OS (HR = 1.387, 95% CI = 1.034–1.860, P = 0.029). We then constructed a prognostic nomogram integrating SIRT2 and clinical variables to predict 1-, 3-, and 5-year OS probabilities (**Figure 5H**). To evaluate the predictive performance of this nomogram, we generated time-dependent ROC curves for long-term survival prediction (**Supplementary Figure 1B**), which yielded AUC values of 0.629, 0.633, and 0.625, indicating moderate capacity to stratify patients with distinct survival risks. Calibration curves validated model accuracy by showing strong alignment between observed survival fractions and nomogram-predicted probabilities (**Supplementary Figure 1C**). Collectively, these results support SIRT2 as an independent prognostic biomarker for LUAD.

### 3.5 Correlation between SIRT2 genomic alterations and clinical outcomes in LUAD

**Figure 6:**
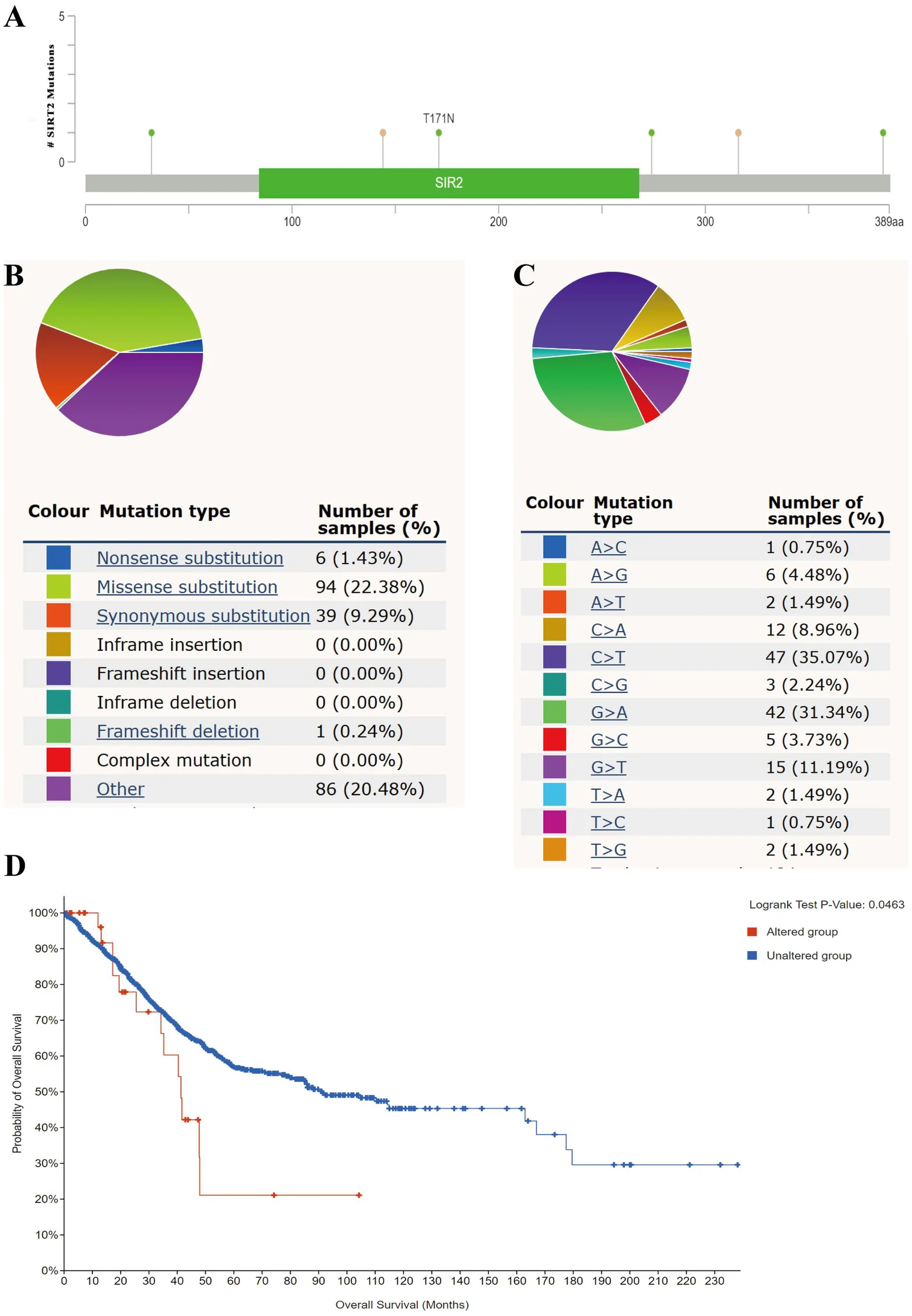
Mutation analysis of SIRT2 in LUAD. **(A)** Distribution of SIRT2 mutations in LUAD from cBioPortal. A total of 30 patients harbored SIRT2 genomic alterations, among which only 3 carried missense mutations. **(B, C)** Classification of SIRT2 mutation types based on COSMIC. **(D)** Kaplan-Meier survival analysis comparing overall survival between SIRT2-altered (n=30) and SIRT2-unaltered (n=465) LUAD patients.

Because somatic mutations in cancer-related genes are often linked to adverse clinical outcomes (32–34), the mutation profile of SIRT2 in LUAD was examined using the cBioPortal platform. SIRT2 alterations were identified in approximately 10% of LUAD cases within the cBioPortal Firehose Legacy TCGA-LUAD cohort, with a total of 30 patients carrying SIRT2 genomic alterations, among whom only 3 harbored missense variants (**Figure 6A**). Although survival analyses on cBioPortal suggested that SIRT2 genetic alterations may correlate with LUAD prognosis (**Figure 6D**), this result lacks statistical reliability due to the extremely small number of mutated patients and requires validation in larger independent cohorts. Mutation patterns were further evaluated using the COSMIC database. Missense mutations accounted for approximately 22.38% of all SIRT2 variants (**Figure 6B**), and among all base substitutions, C > T transitions were the most common, representing 35.07% of events (**Figure 6C**).

### 3.6 Functional analyses of SIRT2-associated genes in LUAD

**Figure 7:**
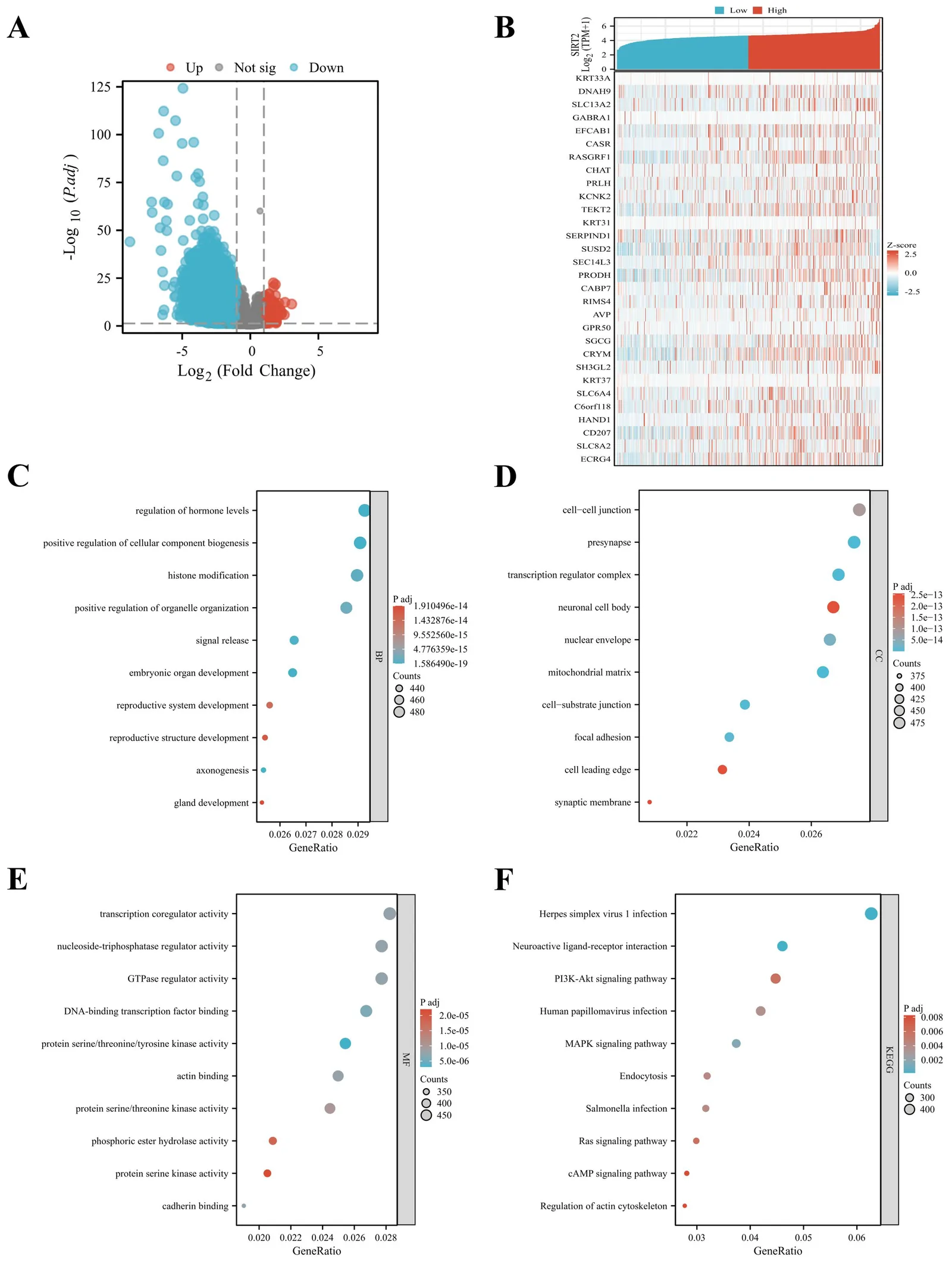
Functional enrichment of SIRT2-associated genes in LUAD. **(A)** Volcano plot of DEGs. **(B)** Heatmap showing the top 30 genes positively correlated with SIRT2. **(C–F)** GO and KEGG enrichment analyses of SIRT2-related genes.

Differential expression analysis of SIRT2 in the TCGA-LUAD dataset was undertaken with limma in R, using thresholds of |logFC| > 1 and adjusted p < 0.05 (**Figure 7A**). Correlation analysis identified 30 genes positively correlated with SIRT2 and 7,127 that were negatively associated (Pearson r > 0.3, p < 0.05). The top 30 genes showing positive association are illustrated in **Figure 7B**.

A total of 3,481 SIRT2-associated genes underwent GO and KEGG analyses using the ClusterProfiler in R. To control for multiple testing, p-values underwent FDR adjustment, and only terms with p < 0.05 and Q < 0.05 represented significance. These genes were enriched in 2,178 biological processes, 324 cellular components, 99 molecular functions, and 58 KEGG pathways. The top 10 enriched terms in each GO category and KEGG pathway were visualized using bubble plots (**Figures 7C–F**). GO annotations suggested involvement in the regulation of hormone levels, positive regulation of cellular component biogenesis, signal release, mitochondrial matrix organization, transcription regulator complexes, cell-cell junctions, protein serine/threonine/tyrosine kinase activity, DNA-binding transcription factor binding, spindle assembly, GTPase regulator activity, and protein serine kinase activity (**Figures 7C–E**). KEGG pathway analysis highlighted associations with neuroactive ligand-receptor interaction, MAPK signaling, herpes simplex virus 1 infection, Ras signaling, and PI3K-Akt signaling pathways (**Figure 7F**).

### 3.7 Relationship between SIRT2 expression and immune cell infiltration in LUAD

**Figure 8:**
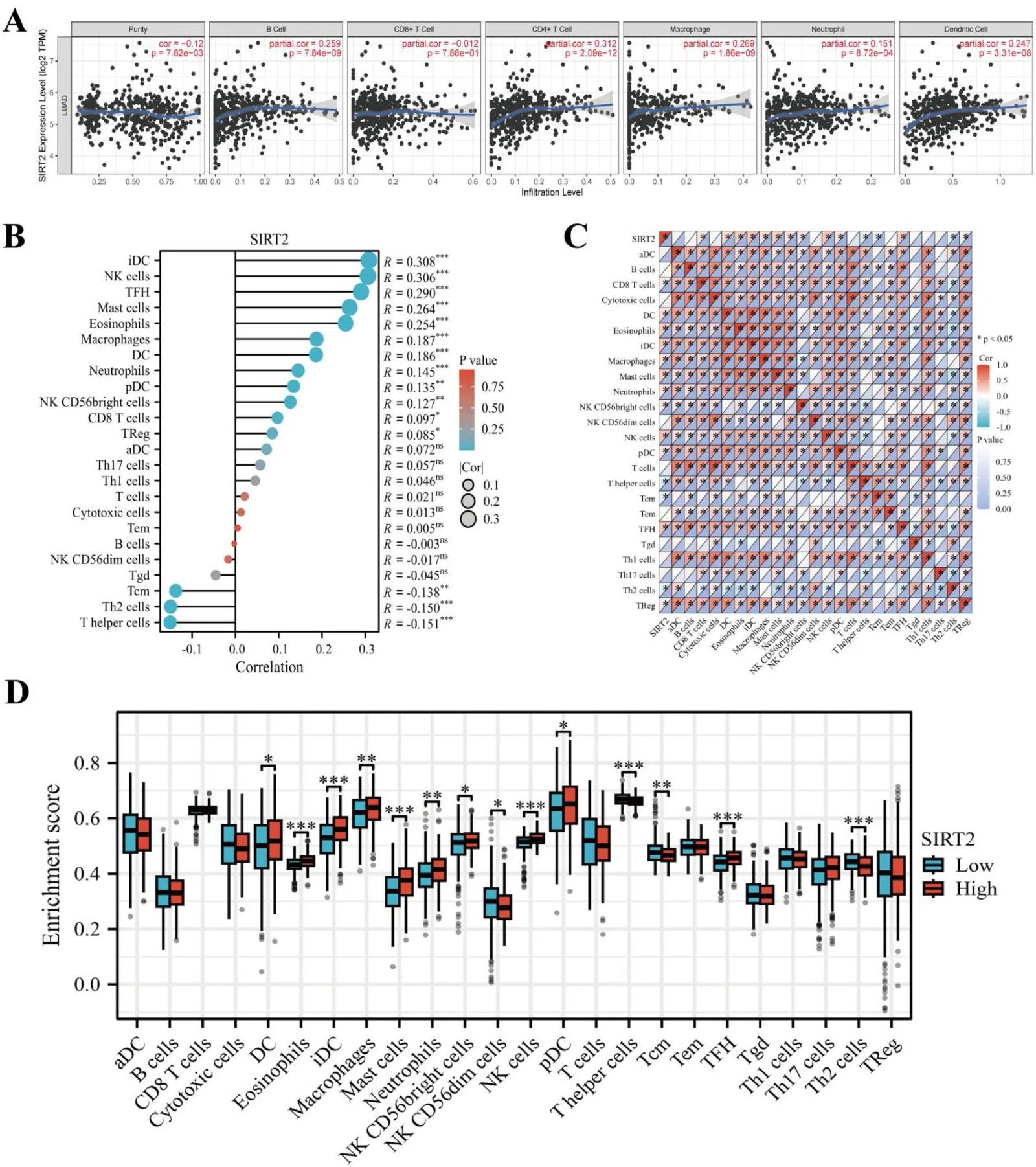
Association between SIRT2 levels and immune infiltration in LUAD. **(A)** Associations between SIRT2 and tumor purity or immune cell subsets analyzed by TIMER 2.0; partial cor denotes partial correlation adjusted for tumor purity. **(B)** Relationships between SIRT2 and infiltrating immune cells in LUAD. **(C)** Heatmap of interactions among infiltrating immune cells. **(D)** Comparison of immune cell proportions between low- and high-SIRT2 expression groups; ssGSEA enrichment scores based on TCGA immune signatures. *p < 0.05; **p < 0.01; ***p < 0.001; ns, not significant.

The association between SIRT2 expression and tumor-infiltrating immune cells was evaluated using the TIMER platform with adjustment for tumor purity. SIRT2 expression in LUAD showed significant correlations with tumor purity (R = −0.12, p = 7.82 × 10^-3^), B cells (R = 0.259, p = 7.84 × 10^-9^), CD4^+^ T cells (R = 0.312, p = 2.09 × 10^-12^), macrophages (R = 0.269, p = 1.86 × 10^-9^), neutrophils (R = 0.151, p = 8.72 × 10^-4^), and dendritic cells (R = 0.247, p = 3.31 × 10^-8^), while no significant association was observed with CD8^+^ T cells (R = −0.012, p = 0.788) (**Figure 8A**). These data suggest that higher SIRT2 expression is statistically correlated with elevated infiltration levels of several immune-effector cell types, which may facilitate antitumor immunity. Conversely, lower SIRT2 expression is associated with reduced estimated infiltration of B cells, CD4^+^T cells and dendritic cells, which may correlate with signatures of immune evasion.

Further analysis using TCGA-based immune deconvolution showed that SIRT2 levels were positively linked with interdigitating dendritic cells (iDCs; R = 0.308, p < 0.001), NK cells (R = 0.306, p < 0.001), and T_FH_ cells (R = 0.290, p < 0.001) (**Figure 8B**). In contrast, negative correlations were observed with central memory T (T_cm_) cells (R = −0.138, p < 0.01), Th2 cells (R = −0.150, p < 0.001), and total T helper cells (R = −0.151, p < 0.001) (**Figure 8B**). A heatmap was generated to illustrate the interrelationships among tumor-infiltrating immune cell populations (**Figure 8C**). Comparisons between low- and high-SIRT2 expression groups further demonstrated significantly higher proportions of NK CD56^dim^ cells, T helper cells, T_cm_ cells, and Th2 cells in the low-expression group (**Figure 8D**). These findings indicate that low SIRT2 expression is associated with an immunosuppressive microenvironment characterized by increased Th2 and Tcm cells, which are known to promote immune evasion and tumor progression. Additional experimental validation is required.

### 3.8 SIRT2 correlates with GPX4 and potential ferroptosis susceptibility in LUAD

**Figure 9:**
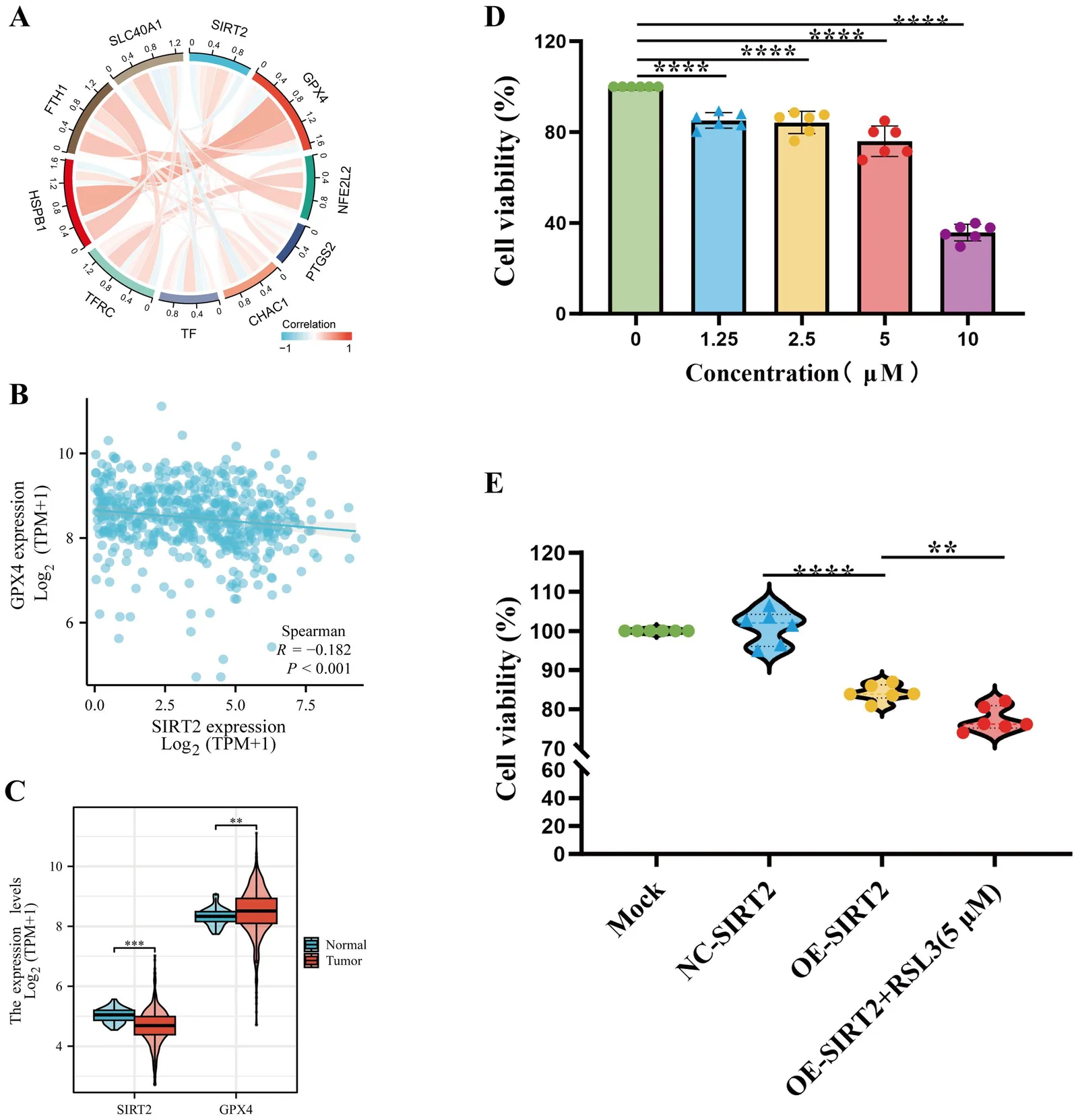
Association between SIRT2 expression and ferroptosis-related markers in LUAD. **(A, B)** Correlations between SIRT2 and ferroptosis-associated genes in the TCGA **(A)** and GEPIA2 **(B)** datasets. **(C)** Comparison of SIRT2 and GPX4 expression between tumor and non-cancerous tissues in TCGA-LUAD. **p < 0.01; ***p < 0.001. **(D)** Viability of A549 cells following 24 h RSL3 treatment assessed by CCK-8 (n = 6, ****p < 0.0001). **(E)** Cell viability after 24 h RSL3 exposure in A549 cells transfected with SIRT2 overexpression plasmid for 24 h, measured by CCK-8 (n = 6, **p < 0.01, ****p < 0.0001).

Prior analyses have demonstrated that suppression of oxidative phosphorylation and reduced intracellular ROS levels can inhibit ferroptosis (35–37). Increasing evidence further suggests that impairment of ferroptotic cell death promotes metastatic progression in various tumors (38–40). On this basis, we postulated that the downregulation of SIRT2 may facilitate LUAD progression by attenuating ferroptosis. To test this hypothesis, Pearson correlations were determined using the GEPIA2 platform and TCGA-LUAD datasets to examine the relationship between SIRT2 expression and key ferroptosis-associated genes. SIRT2 expression was significantly correlated with SLC40A1, PTGS2, TFRC, GPX4, SLC7A11, and NFE2L2 (NRF2), among which the most marked association was observed with GPX4 (R = −0.1820, p < 0.001) (**Figures 9A–C**). GPX4 is a central negative regulator of ferroptosis; its elevated expression inhibits lipid peroxidation and prevents ferroptotic cell death. The negative correlation between SIRT2 and GPX4 suggests that SIRT2 overexpression downregulates GPX4 and aggravates RSL3-induced cell death.

**Figure 10:**
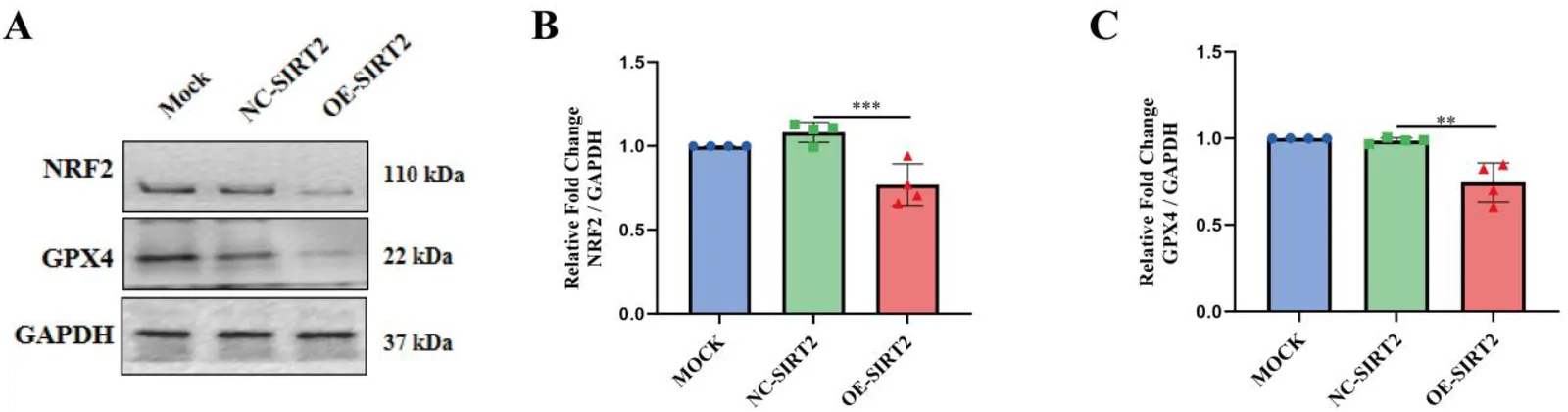
Western blot and quantification of NRF2 and GPX4 in SIRT2-overexpressing A549 cells. **(A)** Representative western blot images of NRF2,GPX4, and GAPDH in A549 cells transfected with SIRT2 overexpression plasmid; (n=4). **(B)** Densitometric quantification of NRF2 protein levels normalized to GAPDH. **(C)** Densitometric quantification of GPX4 protein levels normalized to GAPDH. Mock: blank transfection; NC-SIRT2: empty vector control; OE-SIRT2: SIRT2 overexpression group. **p < 0.01, ***p <0.001.

*In vitro* functional validation was conducted using the ferroptosis inducer RSL3. A dose-dependent reduction in A549 cell viability was observed following RSL3 treatment relative to untreated controls (**Figure 9D**). Notably, enforced SIRT2 expression enhanced RSL3 inhibition of A549 cell viability (**Figure 9E**). To directly test whether SIRT2 regulates GPX4 protein levels, we performed western blotting in A549 cells transfected with SIRT2 overexpression plasmid. As shown in **Figure 10**, SIRT2 overexpression markedly suppressed GPX4 expression relative to control (p < 0.01; **Figures 10A, C**). Densitometric quantification also confirmed a significant decline in NRF2 protein levels upon SIRT2 overexpression (p < 0.001; **Figures 10A, B**). Collectively, these data suggest that SIRT2 expression is associated with GPX4 levels and RSL3-induced cytotoxicity, which may contribute to LUAD progression.

### 3.9 Molecular docking

**Figure 11:**
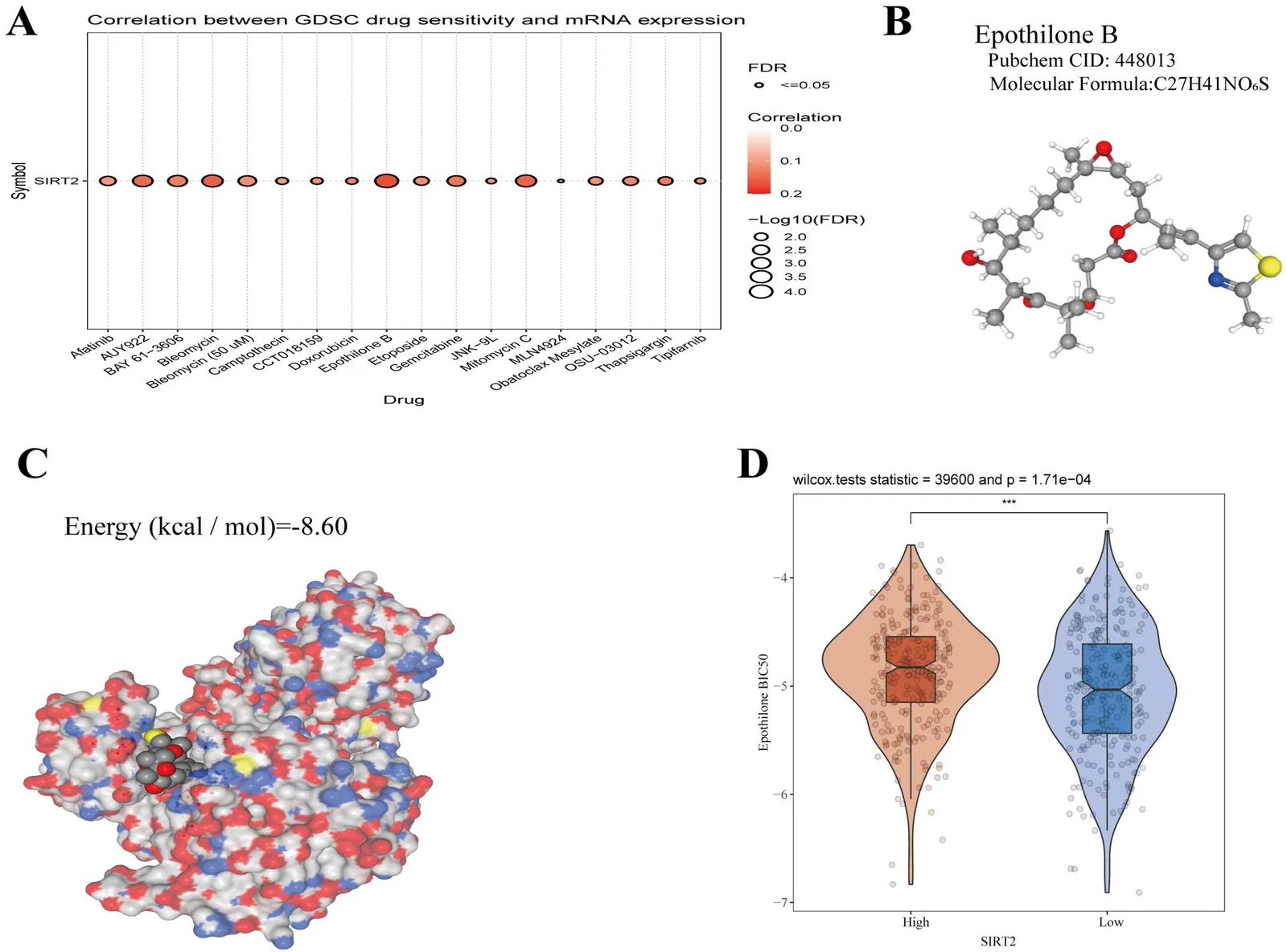
Molecular docking of SIRT2. **(A)** Drug sensitivity of SIRT2 using the GSCA platform. **(B)** Chemical structure of Epothilone **(B, C)** Docking model showing the interaction between Epothilone B and SIRT2. **(D)** Correlation between SIRT2 expression and IC_50_ values for Epothilone B.

Drug sensitivity analysis using the GDSC database identified a significant positive correlation between SIRT2 expression and sensitivity to Epothilone B (cor = 0.17, FDR = 8.19 × 10^-5^) (**Figure 11A**). Because a higher IC50 indicates lower drug sensitivity, this positive correlation implies that elevated SIRT2 expression is associated with relative resistance to Epothilone B. However, this finding prompted further exploration of the molecular interactions between SIRT2 and Epothilone B, as direct binding might still modulate SIRT2 activity regardless of the IC50 direction.

Molecular docking simulations were carried out to predict the binding conformation and affinity between the two molecules. Docking is a computational approach that estimates the most favorable orientation and interaction energy when a ligand binds to a target protein. Binding affinity is assessed according to binding energy, with values below −5 kcal/mol considered indicative of meaningful interaction and values below −7 kcal/mol reflecting substantial binding (41, 42). The docking analysis demonstrated a stable and energetically favorable interaction between SIRT2 and Epothilone B, with a calculated binding energy of −8.60 kcal/mol (**Figures 11B, C**). Importantly, static molecular docking constitutes only an in silico prediction. It cannot verify genuine intracellular binding, nor can it clarify whether Epothilone B activates or inhibits the catalytic activity of SIRT2. Furthermore, static simulations fail to capture dynamic conformational changes within the protein–ligand complex. Therefore, the predicted binding interaction requires validation via biochemical binding assays and SIRT2 deacetylase activity measurements.

Nevertheless, the positive correlation between SIRT2 expression and Epothilone B IC50 implies that tumor cells with higher SIRT2 levels exhibit lower drug sensitivity. Two competing hypotheses may explain this apparent inconsistency: Epothilone B could suppress the tumor-suppressive activity of SIRT2, or it may bind SIRT2 without altering its enzymatic function. Further *in vitro* biochemical experiments, including protein binding detection and SIRT2 deacetylase activity assays, are required to clarify the functional impact of Epothilone B on SIRT2 and illuminate its relevance to drug response (**Figure 11D**).

### 3.10 SIRT2 overexpression inhibits malignant phenotypes in LUAD cells

**Figure 12:**
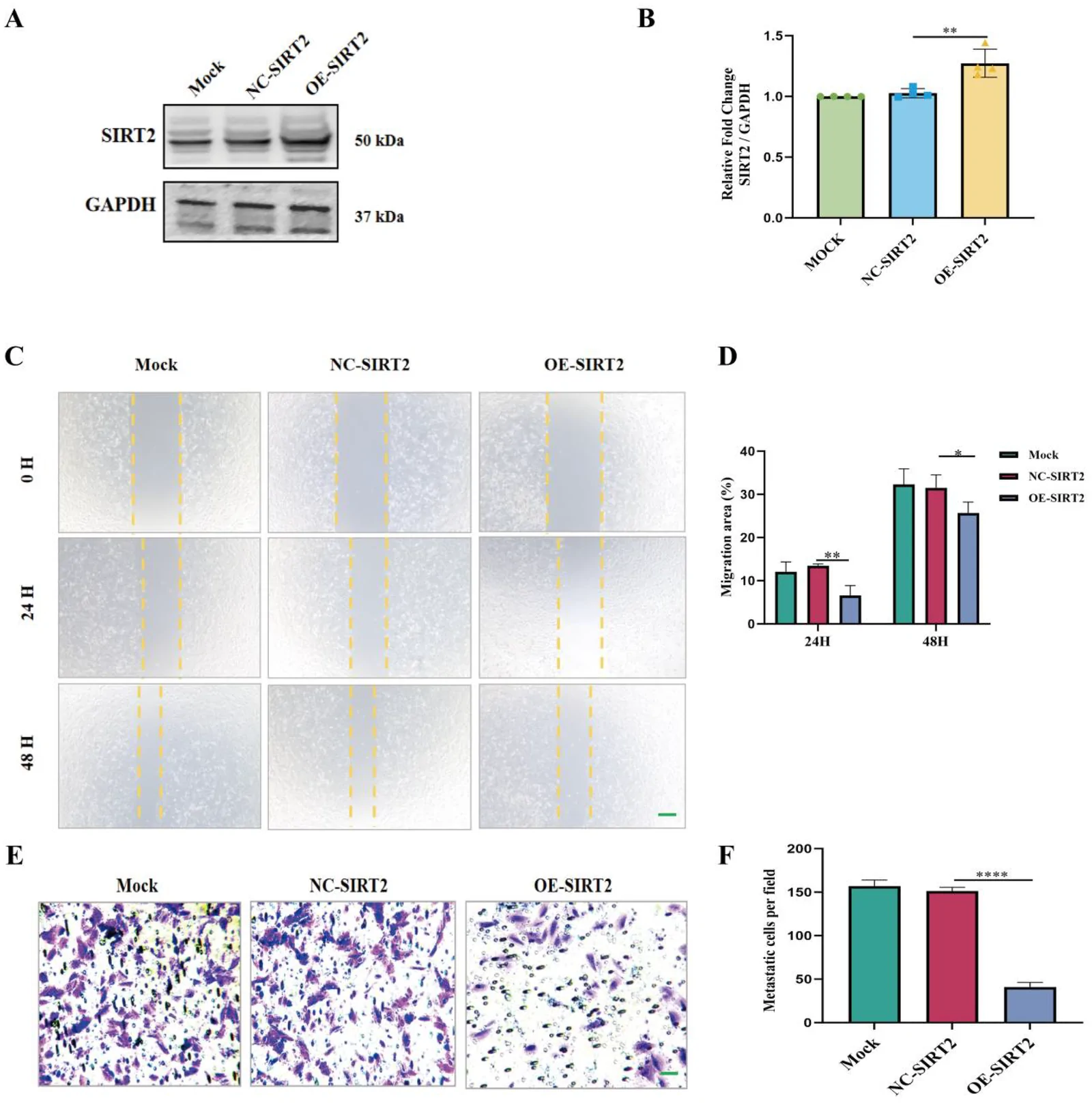
Effects of SIRT2 overexpression on A549 cell migration, and invasion. **(A, B)** Western blotting confirming elevated SIRT2 protein levels following transfection (n = 4). **(C–F)** Elevated SIRT2 expression markedly inhibited cell migration **(C, D)**, and invasion **(E, F)** in A549 cells (n = 4-6). Scale bar = 50 μm. *p < 0.05, **p < 0.01, ****p < 0.0001. Mock: blank transfection; NC-SIRT2: empty vector control; OE-SIRT2: SIRT2 overexpression group.

To experimentally validate the above findings, SIRT2 was ectopically expressed in A549 LUAD cells using an overexpression plasmid. Western blotting confirmed successful upregulation of SIRT2 protein levels (**Figures 12A, B**). Wound healing assays showed a prominent decrease in the migratory capacity of SIRT2-overexpressing cells (**Figures 12C, D**). Moreover, Transwell invasion assays revealed that elevated SIRT2 expression markedly suppressed the invasive potential of A549 cells (**Figures 12E, F**). In summary, these results collectively demonstrate that ectopic overexpression of SIRT2 robustly attenuates multiple malignant behaviors in A549 cells.

## 4 Discussion

LUAD is ranked first in terms of cancer-related mortality, posing a major global health challenge (43). Although recent advances in immunotherapy and molecularly targeted treatments have improved outcomes for some patients, most individuals with LUAD are still diagnosed at advanced stages, which severely limits therapeutic efficacy and contributes to poor overall survival (44). Consequently, the identification of novel biomarkers that can reliably predict prognosis and guide therapeutic strategies is urgently needed. Genetic alterations in the SIRT2 gene have been implicated in multiple pathological conditions (45–47), and aberrant SIRT2 expression has been linked to tumor initiation and progression in gastric cancer (48). However, its expression profile, regulatory mechanisms, and clinical relevance in LUAD have not been systematically explored. In this study, we integrated large-scale bioinformatics analyses with *in vitro* functional experiments to characterize the prognostic value and biological role of SIRT2 in LUAD.

Consistent with our pan-cancer transcriptomic profiling results, SIRT2 exhibits context-dependent dual biological functions across distinct human malignancies. Its expression is drastically suppressed in lung adenocarcinoma, breast cancer and uterine corpus endometrial carcinoma. Our *in vitro* functional tests in A549 cells only confirm that SIRT2 overexpression elicits tumor-suppressive effects by restraining the proliferation and invasive capacity of this cell line and sensitizing it to ferroptosis. By contrast, SIRT2 is overexpressed in cholangiocarcinoma, liver hepatocellular carcinoma and kidney renal clear cell carcinoma, where it activates oncogenic signaling cascades and facilitates disease progression. We propose three primary determinants responsible for such opposing functional phenotypes: organ-specific immune-metabolic microenvironments (49), differential intracellular pools of acetylated substrates available for SIRT2 deacetylation, and heterogeneous driver mutation profiles among distinct tumor molecular subtypes (50). Multiple prior sirtuin-family studies have described this bidirectional regulatory characteristic of SIRT2, whose biological functions are highly context-dependent. Accumulated evidence further proves that SIRT2’s functional role shifts dynamically depending on tumor entity, genetic background and disease stage; a typical example is prostate cancer, where SIRT2 acts as a tumor suppressor in early lesions but acquires oncogenic properties at late disease phases (51).

Our data demonstrated that SIRT2 expression is significantly reduced in LUAD tissues, suggesting that it may function as a tumor suppressor. Importantly, SIRT2 levels were markedly lower in stage T3-T4 tumors than in T1-T2 tumors (p < 0.05), indicating that decreased SIRT2 expression is associated with increased tumor aggressiveness and metastatic potential. Functional assays further supported this conclusion, as forced SIRT2 overexpression in A549 cells significantly inhibited cellular proliferation, migration, and invasion. These findings directly confirm the suppressive effect of SIRT2 on malignant phenotypes in LUAD. Consistent with earlier reports identifying SIRT2 as a prognostic indicator in liver cancer (52), survival analysis showed that lower levels of SIRT2 were strongly associated with unfavorable OS, PFI, and DSS. These associations remained stable across multiple clinical subgroups. Based on these results, we constructed a prognostic nomogram incorporating SIRT2 expression, which may serve as a clinically useful tool for risk stratification and outcome prediction in LUAD. Nevertheless, prospective clinical trials are required to validate the nomogram’s utility in real-world settings, and the integration of SIRT2 with existing biomarkers (e.g., PD-L1, EGFR mutations) should be explored to refine patient selection for targeted or immunotherapies. Collectively, these results demonstrate the potential of SIRT2 in the management of the disease.

To further elucidate the functions of SIRT2, GO and KEGG analyses were conducted on SIRT2-related genes. These analyses revealed significant enrichment in pathways related to protein serine kinase activity, DNA-binding transcription factor interactions, and other key regulatory processes. Aberrant activation of protein serine kinases is a hallmark of tumorigenesis and contributes to the regulation of cancer cell proliferation, migration, and survival (53, 54). Notably, SIRT2 has been reported to influence resistance to targeted therapy in colon cancer through modulation of the RTK-RAS-MAPK signaling pathway (55). These observations suggest that SIRT2 may also participate in LUAD pathogenesis by regulating similar oncogenic signaling networks, providing a foundation for future mechanistic studies.

Given the established role of immunotherapy in LUAD treatment (56, 57), we next examined the link between SIRT2 levels and immune cell infiltration. Our analysis showed that SIRT2 levels were significantly correlated with the proportions of numerous immune cell types in the tumor microenvironment. High SIRT2 expression was positively linked with greater infiltration of B cells, CD4^+^ T cells, macrophages, and dendritic cells, as well as higher stromal scores. In contrast, lower SIRT2 levels were associated with higher proportions of NK CD56^dim^ cells, helper T cells, T_cm_, and Th2 cells, which are documented to be linked to immune evasion and the establishment of an immunosuppressive microenvironment. These results show that SIRT2 expression level is statistically correlated with the infiltration abundance of multiple immune cell subsets, which indicates a potential link between SIRT2 and the composition of tumor immune microenvironment. Reduced SIRT2 expression may correlate with impaired immune surveillance and accelerated tumor progression, highlighting its potential relevance to immunotherapeutic strategies for LUAD. Specifically, accumulated Th2 cells could drive M2 macrophage polarization and inhibit cytotoxic T cell function, which may contribute to tumor immune escape. Our findings suggest that SIRT2 may be linked to immune cell recruitment and function, potentially shaping the tumor immune microenvironment.

Ferroptosis has recently gained attention as a promising anticancer mechanism, particularly for tumors that are resistant to conventional therapies (58). Several tumor suppressors, including p53, fumarase, and BAP1, have been shown to enhance cellular sensitivity to ferroptosis (59). In the present study, SIRT2 expression was significantly correlated with multiple ferroptosis-related genes, including GPX4, SLC7A11, and TFRC. Among these, the negative correlation with GPX4 was the most pronounced (R = −0.1820, p < 0.001). GPX4 is a central regulator of ferroptosis, and its elevated expression suppresses lipid peroxidation, thereby preventing ferroptotic cell death (60). Cellular experiments further demonstrated that SIRT2 overexpression enhanced the cytotoxic effect of the ferroptosis inducer RSL3 in A549 cells. Moreover, our experimental data revealed that SIRT2 overexpression was accompanied by reduced GPX4 protein levels. These observations suggest that SIRT2 may be linked to increased cellular susceptibility to RSL3 in LUAD cells alongside downregulated GPX4, which could contribute to restraining tumor growth and progression. Notably, SIRT2 overexpression downregulated NRF2 in A549 cells, conflicting with the weak SIRT2-NRF2 correlation in TCGA-LUAD, possibly due to heterogeneous clinical tumor microenvironments. This appears to be the first investigation showing a link between SIRT2 and the regulation of ferroptosis in LUAD, offering a novel therapeutic perspective for combination treatment strategies.

In addition, molecular docking analysis revealed a strong binding interaction between SIRT2 and Epothilone B, with a binding energy of −8.60 kcal/mol. Per standard docking cutoffs, binding energy values below −7 kcal/mol represent strong ligand-protein affinity; the −8.60 kcal/mol score predicts a potentially stable interaction. Furthermore, SIRT2 levels were positively linked with the IC_50_ of Epothilone B, indicating that higher SIRT2 expression correlates with reduced sensitivity to this agent. Epothilone B is a microtubule-stabilizing agent with documented antitumor activity across a range of malignancies (61–63). Our findings suggest that Epothilone B may directly interact with SIRT2, potentially modulating its biological function, but we emphasize that these docking outcomes are only preliminary computational findings and lack verification via molecular dynamics (MD) simulations and biochemical binding assays. MD simulations would offer comprehensive insights into the dynamic binding stability and conformational kinetics of the SIRT2-Epothilone complex, and such validations will be performed in our follow-up independent research. The clinical relevance of this predicted interaction requires further experimental investigation given the observed drug resistance association.

Despite these promising results, there are several limitations. First, many conclusions are derived from bioinformatics analyses and *in vitro* experiments, underscoring the need for validation in large, independent clinical cohorts. Second, the present study did not evaluate the link between SIRT2 expression and key LUAD molecular features such as KRAS, TP53, or EGFR mutations due to the lack of corresponding mutation data in our experimental cohort. This represents an important limitation, as such associations could provide deeper insights into SIRT2’s role in LUAD pathogenesis and should be addressed in future studies. Third, we did not perform *in vivo* experiments such as xenograft or orthotopic models to validate the tumor-suppressive role of SIRT2 in an intact immune and stromal microenvironment. This limits the translational relevance of our study, and such investigations are strongly warranted. Fourth, SIRT2 function is NAD^+^-dependent, yet we used mRNA expression and protein levels as surrogates for enzymatic activity; future studies should measure deacetylase activity directly. Fifth, immune infiltration estimates from deconvolution algorithms cannot distinguish spatial distributions of immune cells within the tumor microenvironment; spatial transcriptomics or multiplex IHC would provide deeper insights. Sixth, it should be noted that the correlations observed between SIRT2 expression and immune infiltration or ferroptosis-related genes do not imply causation. Computational correlation results based on bulk transcriptome deconvolution cannot verify the direct regulatory effects of SIRT2 on immune cells. Besides, we did not analyze the correlation of SIRT2 with immune checkpoint genes and cytolytic activity signatures in this work, which limits the comprehensive evaluation of anti-tumor immune status. The mechanistic basis underlying these associations requires further experimental validation through functional studies, including loss- and gain-of-function approaches in appropriate *in vivo* models. In particular, our ferroptosis-related evidence is indirect and inadequate: we only provided GPX4 western blot and RSL3 cell viability data, without detecting lipid ROS, MDA, ferrous ion or performing ferrostatin-1 rescue tests, which weakens the reliability of ferroptosis-related deductions. All *in vitro* functional experiments were only performed in A549 cells without SIRT2 knockdown reverse validation or verification in other LUAD cell lines, restricting the generalizability of our cellular results. Finally, the molecular mechanisms underlying SIRT2-mediated regulation of immune infiltration and ferroptosis remain to be fully elucidated.

In summary, this study demonstrates that SIRT2 is significantly downregulated in LUAD and that its levels show close associations with tumor progression, immune microenvironment remodeling, and GPX4-mediated ferroptosis regulation. SIRT2 exerts antitumor effects by suppressing cell proliferation, migration, and invasion, while also modulating antitumor immunity and ferroptotic pathways. Moreover, the direct interaction between SIRT2 and Epothilone B highlights a potential avenue for targeted therapy, though the direction of functional modulation needs clarification. Further *in vivo* and clinical research is warranted to clarify the regulatory mechanisms of SIRT2 and to support the development of personalized diagnostic and therapeutic strategies for LUAD.

## Data Availability

The original contributions presented in the study are included in the article/**Supplementary Material**. Further inquiries can be directed to the corresponding authors.

## References

[1] Song Y Kelava L Kiss I . MiRNAs in lung adenocarcinoma: Role, diagnosis, prognosis, and therapy. Int J Mol Sci. (2023) 24:13302. doi: 10.3390/ijms241713302 37686110 PMC10487838

[2] Liu M Gu L Zhang Y Li Y Zhang L Xin Y . LKB1 inhibits telomerase activity resulting in cellular senescence through histone lactylation in lung adenocarcinoma. Cancer Lett. (2024) 595:217025. doi: 10.1016/j.canlet.2024.217025 38844063

[3] Haggstrom L Chan WY Nagrial A Chantrill LA Sim H Yip D . Chemotherapy and radiotherapy for advanced pancreatic cancer. Cochrane Database Systematic Rev. (2024) 12:CD11044. doi: 10.1002/14651858.CD011044.pub3 39635901 PMC11619003

[4] Girnyi S Marano L Skokowski J Mocarski P Kycler W Gallo G . Prehabilitation approaches for gastrointestinal cancer surgery: A narrative review. Rep Pract Oncol Radiotherapy J GreatPoland Cancer Center Poznan Polish Soc Radiat Oncol. (2024) 29:614–26. doi: 10.5603/rpor.103136 39759553 PMC11698552

[5] Gao S Zhang Z Ye K Wang W Li J Xu T . Comprehensive characterization of Rubus idaeus L. polysaccharides: Extraction, purification, structural diversity, biological efficacy, and structure-activity relationships. J Ethnopharmacol. (2026) 355:120677. doi: 10.1016/j.jep.2025.120677 41043662

[6] Jiang Y Wang C Zu C Rong X Yu Q Jiang J . Synergistic potential of nanomedicine in prostate cancer immunotherapy: Breakthroughs and prospects. Int J Nanomedicine. (2024) 19:9459–86. doi: 10.2147/IJN.S466396 39371481 PMC11456300

[7] Gao S Ye K Zhang Z Wang W Li J Xu T . Polysaccharides of Pseudostellaria heterophylla (Miq.) Pax ex Pax et Hoffm: Extraction, purification, structural characteristics, pharmacological activities, and structure-activity relationships: A review. Int J Biol Macromol. (2025) 330:148082. doi: 10.1016/j.ijbiomac.2025.148082 41046896

[8] Haga Y Sakamoto Y Kajiya K Kawai H Oka M Motoi N . Whole-genome sequencing reveals the molecular implications of the stepwise progression of lung adenocarcinoma. Nat Commun. (2023) 14:8375. doi: 10.1038/s41467-023-43732-y 38102134 PMC10724178

[9] Cui Y Li J Zhang P Yin D Wang Z Dai J . B4GALT1 promotes immune escape by regulating the expression of PD-L1 at multiple levels in lung adenocarcinoma. J Exp Clin Cancer Res Cr. (2023) 42:146. doi: 10.1186/s13046-023-02711-3 37303063 PMC10259029

[10] Murphy SJ Wigle DA Lima JF Harris FR Johnson SH Halling G . Genomic rearrangements define lineage relationships between adjacent lepidic and invasive components in lung adenocarcinoma. Cancer Res. (2014) 74:3157–67. doi: 10.1158/0008-5472.CAN-13-1727 24879567 PMC4399556

[11] Robles Gómez A Oliva Lozano J Rodríguez Fernández P Ruiz González E Tilve Gómez A Arenas-Jiménez J . Lung adenocarcinoma: Characteristic radiological presentations. Radiologia. (2024) 66:542–54. doi: 10.1016/j.rxeng.2024.11.003 39674619

[12] Jiang H Bu L . Progress in the treatment of lung adenocarcinoma by integrated traditional Chinese and Western medicine. Front Med (Lausanne). (2024) 10:1323344. doi: 10.3389/fmed.2023.1323344 38259856 PMC10802683

[13] Hutchinson BD Shroff GS Truong MT Ko JP . Spectrum of lung adenocarcinoma. Semin Ultrasound Ct Mr. (2019) 40:255–64. doi: 10.1053/j.sult.2018.11.009 31200873

[14] Zhang H Luo M Feng J Tan J Jiang Y Frishman D . Spatial features of tumor-infiltrating lymphocytes in primary lesions of lung adenocarcinoma predict lymph node metastasis. J Transl Med. (2025) 23:842. doi: 10.1186/s12967-025-06860-1 40713757 PMC12297629

[15] Qi Y Yang L Liu B Liu L Liu Y Zheng Q . Highly accurate diagnosis of lung adenocarcinoma and squamous cell carcinoma tissues by deep learning. Spectrochimica Acta Part A Mol Biomolecular Spectrosc. (2022) 265:120400. doi: 10.1016/j.saa.2021.120400 34547683

[16] Pun R Kumari N Monieb RH Wagh S North BJ . BubR1 and SIRT2: Insights into aneuploidy, aging, and cancer. Semin Cancer Biol. (2024) 106-107:201–16. doi: 10.1016/j.semcancer.2024.10.005 39490401 PMC11625622

[17] Kaya SG Eren G . Selective inhibition of SIRT2: A disputable therapeutic approach in cancer therapy. Bioorg Chem. (2024) 143:107038. doi: 10.1016/j.bioorg.2023.107038 38113655

[18] Jessop E Young N Garcia-Del-Valle B Crusher JT Obara B Karakesisoglou I . SIRT2 inhibition by AGK2 promotes perinuclear cytoskeletal organisation and reduces invasiveness of MDA-MB-231 triple-negative breast cancer cells in confined *in vitro* models. Cells. (2024) 13:2023. doi: 10.3390/cells13232023 39682770 PMC11639776

[19] Wang B Ye Y Yang X Liu B Wang Z Chen S . SIRT2-dependent IDH1 deacetylation inhibits colorectal cancer and liver metastases. EMBO Rep. (2020) 21:e48183. doi: 10.15252/embr.201948183 32141187 PMC7132198

[20] Wu A Li S Feng C He R Wu R Hu Z . Fn14 controls the SIRT2-mediated deacetylation of Slug to inhibit the metastasis of epithelial ovarian cancer. Advanced Sci (Weinheim Baden-Wurttemberg Germany). (2025) 12:e2501552. doi: 10.1002/advs.202501552 40344622 PMC12279162

[21] Liu K Ma J Ao J Mu L Wang Y Qian Y . The oncogenic role and immune infiltration for CARM1 identified by pancancer analysis. J Oncol. (2021) 2021:2986444. doi: 10.1155/2021/2986444 34745258 PMC8566078

[22] Chen H Ao Q Wang Y Qian Y Cheng Q Zhang W . SOX11 as a potential prognostic biomarker in hepatocellular carcinoma linked to immune infiltration and ferroptosis. Chin J Cancer Res. (2024) 36:378–97. doi: 10.21147/j.issn.1000-9604.2024.04.03 39246708 PMC11377886

[23] Ou G Lin W Zhao W . Construction of long noncoding RNA-associated ceRNA networks reveals potential biomarkers in Alzheimer's disease. J Alzheimer's Dis Jad. (2021) 82:169–83. doi: 10.3233/JAD-210068 34024830

[24] Zhao WJ Ou GY Lin WW . Integrative analysis of neuregulin family members-related tumor microenvironment for predicting the prognosis in gliomas. Front Immunol. (2021) 12:682415. doi: 10.3389/fimmu.2021.682415 34054873 PMC8155525

[25] Kanehisa M Goto S . KEGG: Kyoto encyclopedia of genes and genomes. Nucleic Acids Res. (2000) 28:27–30. doi: 10.1093/nar/28.1.27 10592173 PMC102409

[26] Ma Y Zhou W Qian Y Mu Y Zhang W . SOX13 as a potential prognostic biomarker linked to immune infiltration and ferroptosis inhibits the proliferation, migration, and metastasis of thyroid cancer cells. Front Immunol. (2024) 15:1478395. doi: 10.3389/fimmu.2024.1478395 39726600 PMC11670200

[27] Zhang W Qiao X Li Q Cui C Qiao C Shen Y . Comprehensive pan-cancer analysis of TRPM8 in tumor metabolism and immune escape. Front Oncol. (2022) 12:914060. doi: 10.3389/fonc.2022.914060 35847920 PMC9281503

[28] Liu CJ Hu FF Xia MX Han L Zhang Q Guo AY . GSCALite: A web server for gene set cancer analysis. Bioinformatics. (2018) 34:3771–2. doi: 10.1093/bioinformatics/bty411 29790900

[29] Zhang W Dao J Li Q Liu C Qiao C Cui C . Neuregulin 1 mitigated prolactin deficiency through enhancing TRPM8 signaling under the influence of melatonin in senescent pituitary lactotrophs. Int J Biol Macromol. (2024) 275:133659. doi: 10.1016/j.ijbiomac.2024.133659 38969045

[30] Vijayakumar S Manogar P Prabhu S Sanjeevkumar SR . Novel ligand-based docking; molecular dynamic simulations; and absorption, distribution, metabolism, and excretion approach to analyzing potential acetylcholinesterase inhibitors for Alzheimer's disease. J Pharm Anal. (2018) 8:413–20. doi: 10.1016/j.jpha.2017.07.006 30595949 PMC6308024

[31] Wang L Xu P Xie X Hu F Jiang L Hu R . Down regulation of SIRT2 reduced ASS induced NSCLC apoptosis through the release of autophagy components via exosomes. Front Cell Dev Biol. (2020) 8:601953. doi: 10.3389/fcell.2020.601953 33344455 PMC7744594

[32] Lu SH Lui K Qian Y Zhou WY Mu YY Zhang W . Prognostic role of SETDB2 in clear cell renal cell carcinoma: Linking immune infiltration, cuproptosis, and tumor suppression. Cancer Manag Res. (2025) 17:675–92. doi: 10.2147/CMAR.S499771 40166493 PMC11956738

[33] Li J Qu Z Zhu D Lu Y Lu J Wu Z . Pan cancer research reveals the role of PTGDS in tumor suppression and immune regulation. NPJ Precis Oncol. (2025) 9:319. doi: 10.1038/s41698-025-01097-z 41028158 PMC12484698

[34] Katsuya N Sentani K Sekino Y Yamamoto Y Kobayashi G Babasaki T . Clinicopathological significance of intelectin-1 in colorectal cancer: Intelectin-1 participates in tumor suppression and favorable progress. Pathol Int. (2020) 70:943–52. doi: 10.1111/pin.13027 33002285

[35] Wang Y Lv M Zhao W . Research on ferroptosis as a therapeutic target for the treatment of neurodegenerative diseases. Ageing Res Rev. (2023) 91:102035. doi: 10.1016/j.arr.2023.102035 37619619

[36] Dixon SJ Olzmann JA . The cell biology of ferroptosis. Nat Rev Mol Cell Biol. (2024) 25:424–42. doi: 10.1038/s41580-024-00703-5 38366038 PMC12187608

[37] Wei X Li X Hu S Cheng J Cai R . Regulation of ferroptosis in lung adenocarcinoma. Int J Mol Sci. (2023) 24:14614. doi: 10.3390/ijms241914614 37834062 PMC10572737

[38] Klasson TD LaGory EL Zhao H Huynh SK Papandreou I Moon EJ . ACSL3 regulates lipid droplet biogenesis and ferroptosis sensitivity in clear cell renal cell carcinoma. Cancer Metab. (2022) 10:14. doi: 10.1186/s40170-022-00290-z 36192773 PMC9528056

[39] Shen M Cao S Long X Xiao L Yang L Zhang P . DNAJC12 causes breast cancer chemotherapy resistance by repressing doxorubicin-induced ferroptosis and apoptosis via activation of AKT. Redox Biol. (2024) 70:103035. doi: 10.1016/j.redox.2024.103035 38306757 PMC10847378

[40] Talty R Bosenberg M . The role of ferroptosis in melanoma. Pigment Cell Melanoma Res. (2022) 35:18–25. doi: 10.1111/pcmr.13009 34407291

[41] Chen P Xie L Ma L Zhao X Chen Y Ge Z . Prediction and analysis of genetic effect in idiopathic pulmonary fibrosis and gastroesophageal reflux disease. IET Syst Biol. (2023) 17:352–65. doi: 10.1049/syb2.12081 37907428 PMC10725712

[42] Li X Zhou H Ma R Guo W Yang X Li X . Structure of POU2AF1 recombinant protein and it affects the progression and treatment of liver cancer based on WGCNA and molecular docking analysis. Int J Biol Macromol. (2024) 278:134629. doi: 10.1016/j.ijbiomac.2024.134629 39128756

[43] Gillette MA Satpathy S Cao S Dhanasekaran SM Vasaikar SV Krug K . Proteogenomic characterization reveals therapeutic vulnerabilities in lung adenocarcinoma. Cell. (2020) 182:200–25. doi: 10.1016/j.cell.2020.06.013 32649874 PMC7373300

[44] Miyahara N Nii K Benazzo A Hoda MA Iwasaki A Klepetko W . Solid predominant subtype in lung adenocarcinoma is related to poor prognosis after surgical resection: A systematic review and meta-analysis. Eur J Surg Oncol J Eur Soc Surg Oncol Br Assoc Surg Oncol. (2019) 45:1156–62. doi: 10.1016/j.ejso.2019.01.220 30772108

[45] Ye Y Yang K Liu H Yu Y Song M Huang D . SIRT2 counteracts primate cardiac aging via deacetylation of STAT3 that silences CDKN2B. Nat Aging. (2023) 3:1269–87. doi: 10.1038/s43587-023-00486-y 37783815

[46] Alhasaniah AH Alissa M Elsaid FG Alsugoor MH AlQahtani MS Alessa A . The enigmatic role of SIRT2 in the cardiovascular system: Deciphering its protective and detrimental actions to unlock new avenues for therapeutic intervention. Curr Probl Cardiol. (2025) 50:102929. doi: 10.1016/j.cpcardiol.2024.102929 39566866

[47] Taneja A Ravi V Hong JY Lin H Sundaresan NR . Emerging roles of Sirtuin 2 in cardiovascular diseases. FASEB J Off Publ Fed Am Societies For Exp Biol. (2021) 35:e21841. doi: 10.1096/fj.202100490R 34582046

[48] Wang J Wu J Wang L Min X Chen Z . The LINC00152/miR-138 axis facilitates gastric cancer progression by mediating SIRT2. J Oncol. (2021) 2021:1173869. doi: 10.1155/2021/1173869 34697541 PMC8541877

[49] Zhu X Zhang F Wei Y Zhao Y Guo J . Immuno-inflammatory-metabolic interactions in cardiovascular diseases: A review from basic mechanisms to clinical translation. Front Immunol. (2026) 17:1818835. doi: 10.3389/fimmu.2026.1818835 42064066 PMC13124526

[50] Makowski L Chaib M Rathmell JC . Immunometabolism: From basic mechanisms to translation. Immunol Rev. (2020) 295:5–14. doi: 10.1111/imr.12858 32320073 PMC8056251

[51] Lin R Yang Y Wu E Zhou M Wang S Zhang Q . SIRT2 promotes cell proliferation and migration through mediating ERK1/2 activation and lactosylceramide accumulation in prostate cancer. Prostate. (2023) 83:71–81. doi: 10.1002/pros.24437 36082450

[52] Fu G Li S Jiang Z Mao Q Xiong N Li X . PGAM5 deacetylation mediated by SIRT2 facilitates lipid metabolism and liver cancer proliferation. Acta Biochim Biophys Sin (Shanghai). (2023) 55:1370–9. doi: 10.3724/abbs.2023155 37580952 PMC10520483

[53] Chauhan VP Singh SS Chauhan A Brockerhoff H . Phosphatidylinositol 3-kinase: inhibition of intrinsic protein-serine kinase activity by phosphoinositides, and of lipid kinase activity by Mn2+. Biochim Biophys Acta. (1995) 1267:139–44. doi: 10.1016/0167-4889(95)00032-n 7612667

[54] Pan H Xue W Zhao W Schachner M . Expression and function of chondroitin 4-sulfate and chondroitin 6-sulfate in human glioma. FASEB J Off Publ Fed Am Societies For Exp Biol. (2020) 34:2853–68. doi: 10.1096/fj.201901621RRR 31908019

[55] Bajpe PK Prahallad A Horlings H Nagtegaal I Beijersbergen R Bernards R . A chromatin modifier genetic screen identifies SIRT2 as a modulator of response to targeted therapies through the regulation of MEK kinase activity. Oncogene. (2015) 34:531–6. doi: 10.1038/onc.2013.588 24469059

[56] Chen Y Zhou Y Ren R Chen Y Lei J Li Y . Harnessing lipid metabolism modulation for improved immunotherapy outcomes in lung adenocarcinoma. J Immunother Cancer. (2024) 12:e8811. doi: 10.1136/jitc-2024-008811 38977328 PMC11256034

[57] Zhao F Chen M Wu T Ji M Li F . Integration of single-cell and bulk RNA sequencing to identify a distinct tumor stem cells and construct a novel prognostic signature for evaluating prognosis and immunotherapy in LUAD. J Transl Med. (2025) 23:222. doi: 10.1186/s12967-025-06243-6 39987127 PMC11847374

[58] Zhao L Zhou X Xie F Zhang L Yan H Huang J . Ferroptosis in cancer and cancer immunotherapy. Cancer Commun (London England). (2022) 42:88–116. doi: 10.1002/cac2.12250 35133083 PMC8822596

[59] Yin J Meng X Peng L Xie W Liu X He W . Ferroptosis and cancer immunotherapy. Curr Mol Med. (2023) 23:401–9. doi: 10.2174/1566524022666220509124608 35579155

[60] Xie Y Kang R Klionsky DJ Tang D . GPX4 in cell death, autophagy, and disease. Autophagy. (2023) 19:2621–38. doi: 10.1080/15548627.2023.2218764 37272058 PMC10472888

[61] Li Y Sun J Hu X Pan Y Yan W Li Q . Epothilone B induces apoptosis and enhances apoptotic effects of ABT-737 on human cancer cells via PI3K/AKT/mTOR pathway. J Cancer Res Clin Oncol. (2016) 142:2281–9. doi: 10.1007/s00432-016-2236-y 27591861 PMC11819010

[62] Cheng H Huang H Huang G . Synthesis and antitumor activity of epothilone B. Eur J Med Chem. (2018) 157:925–34. doi: 10.1016/j.ejmech.2018.08.055 30149324

[63] Altmann K . Epothilone B and its analogs - a new family of anticancer agents. Mini-Rev Med Chem. (2003) 3:149–58. doi: 10.2174/1389557033405269 12570848

