# Supplementary material for: SIRT2 is associated with prognosis, ferroptosis susceptibility, and immune infiltration in lung adenocarcinoma
Source: Front Oncol. 2026 Sep 11;16:1900448. doi: 10.3389/fonc.2026.1900448 (PMC13612300; doi:10.3389/fonc.2026.1900448)
Supplement: Supplementary file 1 [file Supplementaryfile1.docx]

**SIRT2 is associated with prognosis, ferroptosis susceptibility, and immune infiltration in lung adenocarcinoma**

Wei Zhang^1^, Li-sha Ren^1^, Huan Bai^1^, Ying-ying Mu^2.3^,Yue Qian^4*^, Hongyu Chen^2*^

1. Medical Affairs Department, The Second Affiliated Hospital of Zunyi Medical University, Zunyi, Guizhou, 563000, China
2. Department of Nephrology, the Second Medical Center of PLA General Hospital, National Clinical Research Center for Geriatric Diseases, Beijing 100853, China
3. Department of Pathology, Zunyi Hospital of Traditional Chinese Medicine, Zunyi, 563000, Guizhou, China
4. Department of Pathogen Biology, Guizhou Nursing Vocational College, Guiyang, 550000, Guizhou, China

*Correspondence to:

Yue Qian. Department of Pathogen Biology, Guizhou Nursing Vocational College, Guiyang, 550000, Guizhou, China. Email: [gznvc_qianyue@163.com](mailto:qlcheng64@163.com)

Hongyu Chen. Department of Nephrology, the Second Medical Center of PLA General Hospital, National Clinical Research Center for Geriatric Diseases, Beijing 100853, China. Email: [498540322@qq.com](mailto:498540322@qq.com)

**Figure. S1. Multivariate Cox regression forest plot, time-dependent ROC curves and calibration curves of the prognostic nomogram based on the TCGA-LUAD cohort.** (A) Multivariate Cox analysis of SIRT2 expression and other clinical pathological factors. (B) 1-, 3-, and 5-year time dependent receiver operating characteristic curves of the predictive value of SIRT2 for overall survival. (C) Calibration curves for 1, 3 and 5 years.
